# Cross-Dataset Generalization in Phonocardiogram Murmur Classification: A Benchmark Study of Domain-Conditional Performance and the Role of Multi-Representation Fusion

**DOI:** 10.3390/jcm15208058

**Published:** 2026-10-17

**Authors:** Hüseyin Kutlu, Cemil Çolak

**Affiliations:** 1 Department of Biostatistics and Medical Informatics, Faculty of Medicine, Adıyaman University, 02040 Adiyaman, Türkiye; hkutlu@adiyaman.edu.tr; 2 Department of Biostatistics and Medical Informatics, Faculty of Medicine, İnönü University, 44280 Malatya, Türkiye

**Keywords:** phonocardiogram, heart murmur, cross-dataset generalization, domain shift, deep learning, cardiac auscultation, uncertainty quantification, external evaluation

## Abstract

**Background/Objectives:** Automated phonocardiogram (PCG) analysis is a candidate tool for scaling up cardiac auscultation, but the internal accuracy reported in many studies may not reflect performance when a model is applied to recordings from a different source. This study evaluated how a broad range of classifiers generalize across PCG datasets and examined whether a multi-representation architecture improves cross-dataset transfer. **Methods:** Eleven approaches were trained on the pediatric CirCor DigiScope dataset and evaluated, without target-domain fine-tuning, on two external datasets: a heterogeneous adult/mixed dataset (the PhysioNet/Computing in Cardiology Challenge 2016 database) and a homogeneous five-class open-access heart sound dataset. The approaches spanned classical machine learning (spport vector machine, random forest), standard deep models (1D-CNN, 2D-CNN, ResNet1D, CNN-BiLSTM, and two hybrid networks), image-based backbones (EfficientNet-B0, MaxViT), and a proposed four-branch fusion model (PCG-MambaConformer) combining raw-signal, mel-spectrogram, and continuous wavelet transform (CWT) representations. Threshold-transfer and precision–recall analyses, adversarial domain adaptation, an uncertainty-quantification benchmark, a disease-specific transfer analysis, multi-architecture ensembling, a branch ablation, a label-grouping sensitivity analysis, a reciprocal (reverse-direction) transfer experiment and explainability analyses (feature-space t-SNE with class-separability metrics and Grad-CAM saliency) were also performed. Performance was summarized using the area under the receiver operating characteristic curve (AUROC) with sensitivity and specificity. **Results:** Internal AUROC was similar across deep models (approximately 0.82–0.90) and largely independent of parameter count, whereas classical models were lower (0.67–0.69). On the heterogeneous external dataset, the highest AUROC was 0.738 and none of the nine deep models reached a sensitivity and a specificity both of at least 0.80 at any of the three thresholds examined. On the homogeneous external dataset, PCG-MambaConformer reached an AUROC of 0.985 (sensitivity 0.718, specificity 0.995 at the 0.5 threshold), whereas other models tended to collapse toward either always-normal or always-abnormal predictions. Adversarial adaptation improved performance on the homogeneous target but reduced it on the heterogeneous target. On the heterogeneous target, even an oracle threshold raised balanced accuracy by only 0.022 on average, whereas on the homogeneous target a source-derived threshold produced a balanced operating point only for PCG-MambaConformer (sensitivity 0.863, specificity 0.980). Models trained in the reverse direction (PhysioNet/CinC Challenge 2016 to CirCor) also transferred poorly (AUROC 0.612–0.677). Branch ablation showed that three of the four single-branch configurations collapsed to a one-class operating point and that the full fusion model achieved the highest AUROC, and a feature-space analysis showed that successful transfer was associated with the class-separability of the target domain (silhouette score highest for the homogeneous dataset) rather than with the proximity of dataset centroids. **Conclusions:** In the two external datasets evaluated, successful cross-dataset PCG transfer was associated with the homogeneity of the target domain; because these datasets also differed in population, recording protocol, disease spectrum, class distribution and signal quality, homogeneity cannot be isolated as the sole determinant of transferability. Internal accuracy was a weak indicator of external behavior, model complexity alone did not close the heterogeneous transfer gap, and balanced cross-dataset performance on a homogeneous target was associated with multi-representation fusion. The near-perfect result on the homogeneous target should be read as technical cross-dataset transfer under favorable, highly curated conditions rather than as clinical external validation, whereas the heterogeneous result is the more realistic indication of current limitations. These findings support multi-metric external evaluation on independent data, followed by prospective clinical validation, before any clinical use is considered.

## 1. Introduction

Cardiac auscultation is an inexpensive, non-invasive, and widely available method for the initial assessment of cardiovascular disease. Its diagnostic yield depends heavily on operator skill, and the reliability of unaided auscultation has declined as echocardiography has become more accessible. In a community screening study of asymptomatic older adults, auscultation by experienced practitioners detected valvular heart disease with a sensitivity of only 32% for mild disease and 44% for significant disease [1], indicating that unaided auscultation is a limited screening test in primary care. In low- and middle-income settings, where access to echocardiography and pediatric cardiology is constrained, this gap is even more consequential [2,3,4]. These limitations have motivated interest in automated phonocardiogram (PCG) analysis as a route to consistent, scalable decision support.

The phonocardiogram is the recorded acoustic signal of cardiac activity; the presence, timing, and quality of murmurs carry diagnostic information about valvular and structural conditions. Over the past decade, supervised machine learning and deep learning have been applied extensively to PCG classification, supported by public datasets and community challenges. The PhysioNet/Computing in Cardiology (CinC) Challenge 2016 assembled a large, multi-source heart-sound database and established a standard normal-versus-abnormal benchmark [5,6]. The CirCor DigiScope dataset subsequently provided the largest pediatric heart-sound collection with detailed murmur annotations, enabling research beyond binary detection [7]. Methodological work on these datasets includes segmentation algorithms [8,9], convolutional and recurrent architectures [10,11], attention-based networks [12], and ensemble or self-supervised strategies [13,14]. Reported internal accuracies are frequently high, and recent multimodal and time–frequency fusion models have pushed internal benchmarks further [15,16,17]. Related work has also applied deep learning to detect specific structural lesions such as septal defects from PCG [18], paralleling the broader adoption of artificial intelligence across cardiac imaging and intervention planning, including echocardiographic function assessment and transcatheter valve procedures [19,20]. Across these applications, a recurring expectation is that a novel non-invasive cardiovascular tool should ultimately demonstrate clinically meaningful diagnostic or prognostic stratification rather than technical discrimination alone; myocardial scar detected by pulse-cancelation echocardiography, for example, was independently associated with appropriate defibrillator intervention after myocardial infarction, illustrating that a non-invasive acoustic marker can carry prognostic information beyond classification accuracy [21]. Automated PCG analysis should be held to the same standard, and the present study therefore treats external, multi-metric performance rather than internal accuracy as the relevant technical evidence, which is a necessary but not sufficient precondition for any later assessment of clinical usefulness.

Despite consistently high internal accuracy in these reports, a recurring concern in clinical machine learning is that models often perform worse when applied to data from a new site, device, or population—a problem framed in terms of dataset shift and limited external validity [22]. This has been documented across imaging domains, where pre-analytical differences between training and test sites degrade performance unless explicitly addressed [23], and external validation is increasingly emphasized as a condition for clinical credibility [24]. Deep learning has reached expert-level performance on some cardiac tasks, such as arrhythmia detection from the electrocardiogram [25,26], and transfer learning is widely used to mitigate data scarcity [27]; nonetheless, for PCG, differences in recording equipment, patient age, acoustic environment, and disease prevalence between datasets create a comparable transfer risk, and most published models are assessed only by internal cross-validation. The degree to which PCG classifiers retain performance under cross-dataset transfer, and whether particular architectures or signal representations are more robust, has received limited systematic attention.

A further consideration for deployment is the calibration of model confidence. Uncertainty-aware models that abstain on low-confidence cases can improve reliability, and uncertainty estimates have been reported to remain informative under domain shift in some imaging settings [28]. Whether such estimates behave reliably for out-of-distribution PCG recordings is an open and clinically relevant question.

The present study addresses these gaps with a systematic cross-dataset benchmark. A single internal dataset (CirCor [7]) was used for training, and two external datasets with contrasting characteristics were used for zero-shot evaluation: a heterogeneous, multi-source adult/mixed dataset (PhysioNet/CinC 2016 [5,6]) and a homogeneous, single-protocol five-class dataset (Yaseen 2018 [29]). The aims were threefold: (a) to compare internal and external performance across eleven approaches spanning classical machine learning, standard deep models, image-based backbones, and a proposed multi-representation fusion model; (b) to test whether adversarial domain adaptation, multi-architecture ensembling, and uncertainty quantification mitigate the transfer gap; and (c) to characterize, through ablation, disease-specific analysis, and explainability methods (feature-space visualization and saliency mapping), the conditions under which cross-dataset generalization succeeds or fails.

## 2. Materials and Methods

### 2.1. Datasets

Three publicly available PCG datasets were used. The CirCor DigiScope dataset served as the internal (training) source. It comprises pediatric heart-sound recordings collected at four primary auscultation locations, with expert murmur annotations [7]. The cross-validation training set comprised 2531 recordings from 753 patients (19 460 five-second segments), with a record-level murmur distribution of 1906 absent, 494 present, and 131 unknown recordings (Table S1). Recordings were segmented into fixed-length segments for model training, and the three murmur labels were grouped into the binary categories used here: present and unknown were treated as abnormal, and absent as normal. The unknown label (131 recordings, 5.2% of the training set) denotes cases in which the annotator could not confidently exclude a murmur; grouping these with the abnormal class is the conservative choice for a screening task, in which failing to flag a possible murmur is the more costly error. Because the two external datasets carry only binary labels, this grouping affects training alone; a sensitivity analysis in which two representative models were retrained after excluding the unknown recordings is reported in Section 3.6 and Supplementary Table S2. The PhysioNet/CinC 2016 dataset was used as a heterogeneous external set; it aggregates multiple sub-databases recorded with different equipment in different environments, predominantly from adults, with normal and abnormal labels [5,6]. The Yaseen 2018 dataset was used as a homogeneous external set; it contains 1000 recordings evenly distributed across five classes (normal, aortic stenosis, mitral regurgitation, mitral stenosis, and mitral valve prolapse), recorded under a consistent protocol [29]. For binary cross-dataset evaluation, the four pathological classes of Yaseen were grouped as abnormal and the normal class as normal, yielding 200 normal and 800 abnormal recordings.

All recordings were resampled to a common sampling rate of 2000 Hz, band-pass filtered with a fourth-order zero-phase Butterworth filter (25–400 Hz), clipped for spikes and artifacts at three times the interquartile range, amplitude-normalized per recording (z-score), and divided into fixed-length 5 s segments with a 2.5 s hop (50% overlap). The identical pipeline was applied to all three datasets; the complete parameter list is given in Supplementary Table S3. For each segment, three representations were computed: the normalized raw waveform, a log-mel spectrogram, and a continuous wavelet transform (CWT) scalogram. The two external datasets were never used for training or hyperparameter selection; all external evaluation was zero-shot. The absence of record overlap between the internal and external sets was verified explicitly (Section 3.6).

### 2.2. Models and Representations

Eleven approaches were evaluated. Two classical machine-learning baselines—a support vector machine with a radial basis function kernel (SVM-RBF) and a random forest—were trained on a 63-dimensional feature vector combining mel-frequency cepstral coefficients, discrete wavelet transform band energies, and spectral and temporal descriptors. Standard deep models comprised a one-dimensional convolutional network (1D-CNN), a two-dimensional convolutional network on mel spectrograms (2D-CNN), a deep residual 1D network (ResNet1D), a convolutional bidirectional long short-term memory network (CNN-BiLSTM), and two hybrid networks combining raw and spectrogram inputs (denoted hybrid-MLP and hybrid-KAN). Two strong image-based backbones, EfficientNet-B0 [30] and MaxViT (tiny) [31], were applied to mel-spectrogram inputs [10,12,27].

The proposed model, PCG-MambaConformer, is a four-branch feature-fusion network. One branch processes the raw waveform with a residual InceptionTime-style encoder [32]; a second branch applies a Conformer encoder [33] with dynamic sinusoidal positional encoding to a spectro-temporal representation; a third branch processes the CWT scalogram with a convolutional image encoder; and a fourth branch applies a state-space (Mamba) sequence encoder [34] to the raw signal. The branch outputs are concatenated and passed to a fusion classifier. The use of the CWT representation follows prior PCG work in which wavelet-based time–frequency images supported multi-class heart-sound classification [15]; zero-phase wavelet formulations have also been used for translation-invariant feature extraction in other time-domain signals [35]. The state-space and Conformer components were included to capture long-range temporal structure in addition to local spectral features, following recent applications of state-space models to physiological sequence data [36]. All reported results were obtained with the official Mamba implementation (mamba-ssm 2.3.0); a fallback implementation of the state-space branch was used only for the reproducibility check reported in Section 3.2; the rationale for combining several representations is examined empirically in the ablation analysis (Section 3.7).

### 2.3. Training and Evaluation Protocol

All models were trained only on the CirCor dataset using five-fold patient-disjoint cross-validation, stratified by murmur class. For deep models, class imbalance was addressed with a weighted sampler and class-weighted cross-entropy with mild label smoothing. Internal performance was computed on the held-out validation fold. For external evaluation, the five fold-specific models were applied to each external dataset and their predicted probabilities were averaged (five-fold ensemble), producing a single record-level prediction per recording. No external labels were used at any point in training or model selection.

Throughout this article, “external evaluation” denotes the zero-shot testing of CirCor-trained models on the two independent public datasets, and “cross-dataset transfer” denotes the resulting change in performance from the source to a target dataset. Both terms refer to technical, retrospective evaluation on public benchmark data and do not constitute clinical external validation, which would require prospective recordings from the intended clinical setting and population. An operating point is described as “balanced” when both sensitivity and specificity are at least 0.70, and as “degenerate” (one-class) when either is below 0.10, that is, when almost all recordings are assigned to a single class. These are descriptive technical criteria adopted for reporting only; no clinical performance threshold was prespecified, and no result reported here should be taken to indicate that any model is ready for clinical use.

The primary performance measure was the area under the receiver operating characteristic curve (AUROC). Sensitivity and specificity at a 0.5 decision threshold were reported alongside AUROC because, under cross-dataset shift, a high AUROC can coexist with a strongly imbalanced operating point. Predictions were aggregated to the highest identifier available in each dataset. For the internal CirCor data, segment probabilities were averaged within a recording and then within a patient, so internal performance is reported at the patient level (753 patients), which is the unit used by the original CirCor evaluation. The two external datasets provide no patient identifiers, so segment probabilities were averaged within a recording and external performance is reported at the recording level. Internal AUROC was computed on the pooled out-of-fold cross-validation predictions and external AUROC on the five-fold ensemble predictions. Ninety-five percent confidence intervals for all AUROC values were estimated by stratified bootstrap resampling with 2000 resamples, and confidence intervals for sensitivity and specificity were computed using the Wilson score method. Because a single fixed threshold can be misleading when class prevalence and score calibration differ between the source and the target domain, three complementary operating-point analyses were performed. First, precision–recall curves and their area (average precision, AP) were computed for every model on every dataset as a prevalence-sensitive complement to AUROC, with the class prevalence of each dataset reported as the no-skill reference. Second, a source-derived threshold was obtained by maximizing Youden’s J statistic on the pooled internal out-of-fold predictions and was then applied unchanged to both external datasets, simulating the realistic deployment situation in which no target labels are available. Third, the target-optimal threshold (maximizing Youden’s J on the target dataset itself) was computed as an oracle upper bound that is unattainable in a zero-shot setting. Comparing the fixed 0.5, source-derived and oracle operating points separates a failure of discrimination, in which no threshold yields acceptable sensitivity and specificity, from a failure of threshold transfer, in which the ranking of recordings is preserved but the decision cut-off is misplaced. Paired AUROC values were compared with the DeLong test; for the multi-model contrasts on each external dataset, the Holm–Bonferroni method was used to control the family-wise error rate. Confidence intervals and tests were computed with custom Python (Python Software Foundation, Wilmington, DE, USA) routines (NumPy/SciPy): AUROC confidence intervals by stratified bootstrap resampling, the DeLong test using the fast midrank algorithm of Sun and Xu [37], and Wilson score intervals for sensitivity and specificity; the implementation is available in the project repository.

### 2.4. Domain Adaptation, Uncertainty, and Ensembling

Adversarial domain adaptation was implemented for the CNN-BiLSTM model using a domain-adversarial neural network (DANN) [38] with a gradient-reversal layer, trained with unlabeled target-domain segments. CNN-BiLSTM was chosen as the adaptation backbone for three reasons. It achieved the highest internal AUROC of all evaluated models (0.903) and the highest AUROC on the heterogeneous external target among the models operating on the raw waveform (0.727), not significantly different from the best image backbone (EfficientNet-B0, 0.738; Section 3.3), so it was the most informative baseline for testing whether unsupervised adaptation can improve a model that already transfers comparatively well. It is a single-input architecture, so the gradient-reversal layer operates on one well-defined feature vector; in a four-branch fusion model, adaptation would require either four domain discriminators or alignment of the fused representation alone, which would confound the effect of adaptation with the choice of where it is applied. Finally, its low training cost (approximately 3 min per fold) made the repeated adversarial runs required for two targets feasible. The purpose of this experiment was to characterize the behavior of unsupervised adaptation under homogeneous versus heterogeneous shift rather than to maximize the performance of the proposed model; applying DANN to PCG-MambaConformer is identified as a direction for future work. Four uncertainty-quantification methods were compared on the internal task and under out-of-distribution conditions: deep ensembles, Monte Carlo dropout, an evidential deep-learning head, and a Kolmogorov–Arnold network ensemble. CirCor served as the in-distribution set and PhysioNet/CinC 2016 as the out-of-distribution set. Uncertainty was quantified as the predictive entropy for the deep ensemble, Monte Carlo dropout and Kolmogorov–Arnold network ensemble, and as the Dirichlet uncertainty (vacuity) for the evidential head; out-of-distribution sensitivity was defined as the difference in mean uncertainty between the out-of-distribution and in-distribution inputs. Calibration was summarized by the expected calibration error (ECE), and selective prediction was assessed by accuracy when the 30% most uncertain cases were abstained. Multi-architecture ensembling combined the predicted probabilities of several models by soft voting, under two configurations: all nine deep models (Ensemble-ALL) and a two-model subset comprising CNN-BiLSTM and PCG-MambaConformer (Ensemble-Balanced).

### 2.5. Disease-Specific, Ablation, Explainability, and Reciprocal-Transfer Analyses

To examine which pathologies transfer across datasets, the CNN-BiLSTM model trained on CirCor was evaluated on each Yaseen pathological class separately, reporting per-class sensitivity and class-versus-normal AUROC. To examine the contribution of each branch of the proposed model, an ablation was performed comprising four single-branch configurations and four leave-one-out configurations, each retrained with five-fold cross-validation and evaluated on both external datasets.

Two explainability analyses were performed on PCG-MambaConformer. First, the pre-fusion feature vectors (the concatenated branch outputs before the fusion classifier) were extracted for all three datasets and projected to two dimensions using t-distributed stochastic neighbor embedding (t-SNE [39]; perplexity 30, PCA initialization). To quantify how separable the normal and abnormal classes were in the original (pre-projection) feature space of each dataset, the silhouette score and a linear-separability AUROC (five-fold cross-validated logistic regression) were computed; for reference, the Euclidean distance between dataset centroids in the t-SNE space was also reported. Second, gradient-weighted class activation mapping (Grad-CAM) [40] was applied to a convolutional layer of the CWT branch to identify the time–frequency regions most responsible for correctly classified predictions, computed for representative normal and abnormal recordings from the homogeneous external dataset.

To test whether the observed transfer difficulty was specific to the pediatric-to-adult direction, a reciprocal experiment was performed for the two representative models (CNN-BiLSTM and PCG-MambaConformer). Each model was retrained from scratch on PhysioNet/CinC 2016, using the same architecture, hyperparameters and five-fold cross-validation protocol as in the forward direction, and was then evaluated without fine-tuning on the pediatric CirCor data (patient level) and on Yaseen 2018 (recording level). Because CinC 2016 provides no patient identifiers, the folds were record-disjoint rather than patient-disjoint. Yaseen 2018 was deliberately not used as a training source in either direction, since its five classes are specific valvular lesions and a Yaseen-trained model would be a lesion detector rather than a general abnormality detector, so its transfer to CirCor would measure label mismatch rather than domain shift.

### 2.6. Implementation

Experiments were implemented in Python 3.13 (Python Software Foundation, Wilmington, DE, USA) using PyTorch 2.10.0 (https://pytorch.org) with CUDA 12.8 (https://developer.nvidia.com/cuda-toolkit). Spectral features were computed with librosa 0.11.0 (https://librosa.org) and the continuous wavelet transform with ssqueezepy 0.6.6 (https://github.com/OverLordGoldDragon/ssqueezepy; Morlet wavelet); PyWavelets 1.8.0 (https://pywavelets.readthedocs.io) was used for the wavelet-based features of the classical baselines. The state-space branch used mamba-ssm 2.3.0 (https://github.com/state-spaces/mamba) and the image backbones used timm 1.0.22 (https://github.com/huggingface/pytorch-image-models); classical models used scikit-learn 1.8.0 (https://scikit-learn.org). All software websites listed above were accessed on 25 September 2026. All experiments were run on a single NVIDIA RTX 6000 Ada Generation GPU (48 GB; NVIDIA Corporation, Santa Clara, CA, USA) with 128 GB of system memory and used a fixed base random seed (42) for reproducibility (the members of the deep ensemble and of the Kolmogorov–Arnold network ensemble in the uncertainty benchmark used seeds 42, 142, 242, 342 and 442; Supplementary Table S4). Figures were generated at 300 dpi. The complete preprocessing parameters, training hyperparameters, software versions, and per-dataset record and segment counts are provided in Supplementary Tables S1 and S3–S5. The study was reported in accordance with the TRIPOD+AI statement [41] for the predictive-modeling components and the CLAIM 2024 update [42] for the image-based and saliency components, with the completed reporting maps provided in Supplementary Tables S6 and S7. The complete source code, including model definitions, training and evaluation scripts, and figure generation, is openly available at https://github.com/huseyinKutlu/PCG-CrossDataset (accessed on 25 September 2026) and archived on Zenodo (v1.0.1; https://doi.org/10.5281/zenodo.22957181).

## 3. Results

### 3.1. Internal Versus External Performance

**Figure 1:**
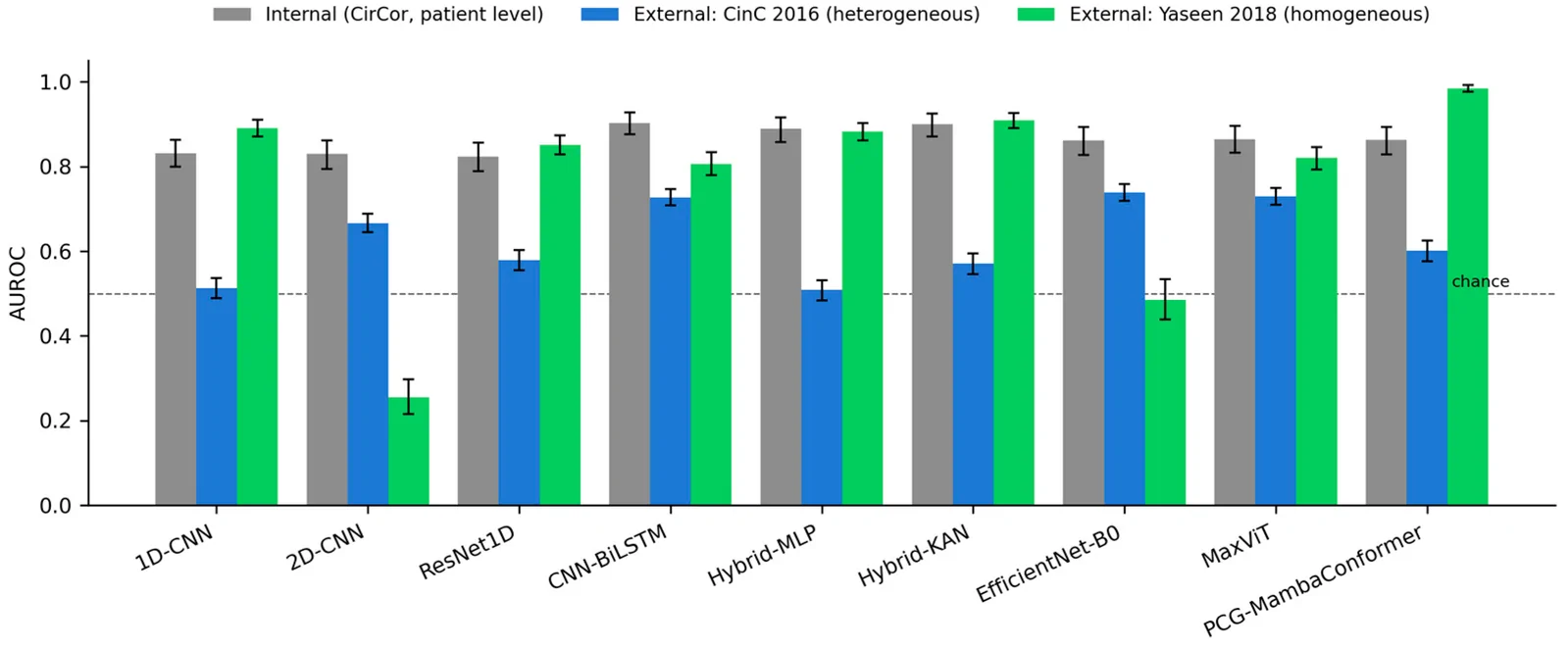
Internal versus external AUROC for the nine deep models, grouped by dataset (internal CirCor [7]; heterogeneous CinC 2016 [5,6]; homogeneous Yaseen 2018 [29]). Error bars denote 95% confidence intervals obtained by stratified bootstrap resampling (2000 resamples). The dashed line denotes chance-level AUROC (0.5). Internal AUROC is computed at the patient level on the pooled out-of-fold cross-validation predictions; external AUROC is computed at the recording level on the five-fold ensemble predictions. The two classical baselines (SVM-RBF, random forest) are reported in the text.

Internal AUROC, computed on the pooled out-of-fold cross-validation predictions, was similar across all deep models, ranging from approximately 0.82 to 0.90, and did not scale with parameter count: the 30-million-parameter MaxViT (0.865; 95% CI 0.832–0.895) did not exceed the approximately 1-million-parameter CNN-BiLSTM (0.903; 95% CI 0.876–0.927). Two classical machine-learning baselines were clearly lower and are reported here for reference: SVM-RBF (internal AUROC 0.670) and random forest (0.690); both failed to transfer, with a sensitivity of zero on the homogeneous external dataset and CinC AUROC of 0.305 and 0.324, respectively. External performance diverged sharply from internal performance and differed between the two external datasets. Table 1 summarizes internal and external AUROC with 95% confidence intervals for the nine deep models, Table 2 reports the corresponding operating points (sensitivity and specificity), and Figure 1 shows the comparison graphically. No internal operating point was degenerate (sensitivity 0.482–0.746, specificity 0.712–0.986; Table 2)—for example, CNN-BiLSTM reached an internal sensitivity of 0.746 (95% CI 0.681–0.802) and specificity of 0.899 (0.872–0.922) and PCG-MambaConformer 0.650 (0.581–0.713) and 0.915 (0.889–0.936)—in contrast to the imbalanced operating points that emerged after transfer, supporting the central observation that internal accuracy was a weak indicator of external behavior.

Two patterns are evident in Table 1 and Table 2. First, internal performance was a weak indicator of external performance: models with similar internal AUROC behaved very differently on the external datasets. Second, the two external datasets produced opposite difficulty profiles. On the heterogeneous CinC dataset, the best AUROC was 0.738 (EfficientNet-B0), and discrimination remained modest for all approaches. On the homogeneous Yaseen dataset, several models exceeded 0.85 AUROC, but most did so with strongly imbalanced sensitivity and specificity—collapsing toward predicting all recordings as normal (for example, Hybrid-KAN, sensitivity 0.006) or all as abnormal (for example, MaxViT, specificity 0.080). The cross-dataset difficulty was therefore not specific to deep models, and a high threshold-independent metric could conceal a degenerate (one-class) operating point.

### 3.2. Balanced Generalization on the Homogeneous Target

**Figure 2:**
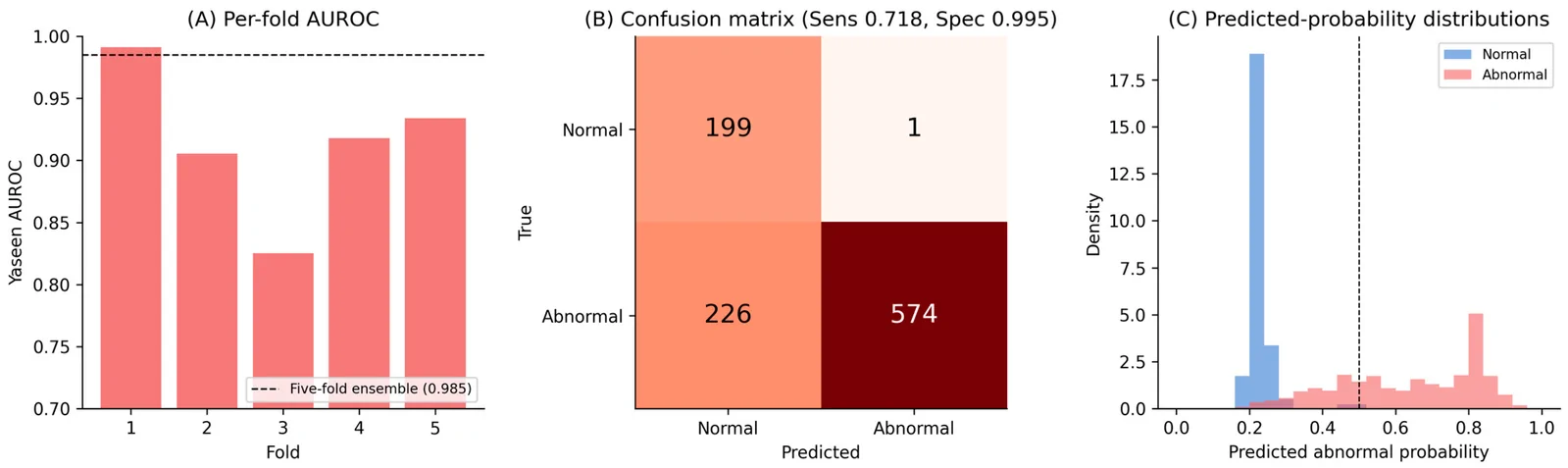
Performance of PCG-MambaConformer on the homogeneous Yaseen dataset: (**A**) per-fold AUROC for folds 1–5, with the five-fold ensemble value shown as a dashed line; (**B**) confusion matrix at the 0.5 threshold (sensitivity 0.718, specificity 0.995), with cell shading proportional to the number of recordings (darker = more recordings); (**C**) predicted-probability distributions for normal (blue) and abnormal (red) recordings; the vertical dashed line marks the 0.5 decision threshold.

Among all approaches, only PCG-MambaConformer achieved a balanced operating point on the homogeneous external dataset, with an AUROC of 0.985 (95% CI 0.977–0.992), sensitivity 0.718 (0.685–0.748), and specificity 0.995 (0.972–0.999). Its Yaseen AUROC was significantly higher than that of every other deep model (DeLong test, all Holm-adjusted *p* < 0.0001), indicating that the advantage was unlikely to reflect sampling variation alone. For this model, external AUROC on Yaseen (0.985) exceeded the internal estimate (0.862), indicating that discrimination was not degraded by transfer to this homogeneous target; the higher external value is best attributed to the curated, high-signal-to-noise nature of the target (Section 4) rather than to superior generalization. Figure 2 shows the per-fold AUROC, the confusion matrix, and the score distribution for this model on Yaseen. The five-fold ensemble was the reported value (AUROC 0.985) and was more stable than any individual fold: per-fold AUROC had a median of 0.918 (interquartile range 0.905–0.934; full range 0.825–0.991), the spread reflecting the limited size of the homogeneous external set (200 normal and 800 abnormal recordings). The confusion matrix showed correct classification of 199 of 200 normal recordings and 574 of 800 abnormal recordings, and normal recordings were concentrated in a narrow band of low predicted probabilities, whereas abnormal recordings were spread over a wide range, with a substantial fraction between 0.3 and 0.5 (mean predicted abnormal probability 0.23 versus 0.64); the ranking was therefore nearly perfect while the 0.5 cut-off lay above part of the abnormal distribution, consistent with the threshold analysis in Section 3.4.

This result was reproducible across two independent implementations of the state-space branch (a fallback implementation and the official implementation), which produced Yaseen AUROC values of 0.972 and 0.985, respectively, supporting that the finding is not an artifact of a single training run.

### 3.3. Heterogeneous Target Remains Unsolved

On the heterogeneous CinC dataset, no method—including the strongest image backbones, the proposed fusion model, domain adaptation, and ensembling—achieved more than modest discrimination. The best single-model AUROC was 0.738 (EfficientNet-B0; 95% CI 0.719–0.758), and the proposed model reached 0.601 (0.576–0.624). The three highest-performing models on CinC (EfficientNet-B0 0.738, MaxViT 0.729, CNN-BiLSTM 0.727) did not differ significantly from one another (DeLong test, all Holm-adjusted *p* > 0.70), indicating that no architecture meaningfully outperformed the others on the heterogeneous target. As described below, neither adversarial adaptation nor multi-architecture ensembling substantially improved discrimination on this dataset. In the reciprocal experiment (Section 2.5; Supplementary Table S8), models trained on PhysioNet/CinC 2016 reached AUROC values of 0.677 (CNN-BiLSTM) and 0.612 (PCG-MambaConformer) on CirCor and 0.926 and 0.942, respectively, on Yaseen 2018; these results are interpreted in Section 4.

### 3.4. Operating-Point Transfer and Threshold Recalibration

The operating points in Table 2 were obtained at a fixed 0.5 decision threshold. Because both score calibration and class prevalence differ between the source and the two targets, this threshold may misrepresent what these models could achieve, and a prevalence-sensitive metric together with an explicit threshold comparison was therefore added (Table 3, Supplementary Figure S1). Because the source-derived and target-optimal thresholds coincide on the internal dataset by construction, the internal operating points are reported separately in Supplementary Table S9.

Precision–recall analysis reproduced the ordering given by AUROC but placed the heterogeneous result in sharper relief. On CinC 2016, where the abnormal prevalence is 0.205, the highest average precision was 0.412 (EfficientNet-B0), closely followed by 0.410 (CNN-BiLSTM), corresponding to an absolute gain of approximately 0.21 over the no-skill baseline; the proposed model reached 0.345. On Yaseen 2018, where the abnormal prevalence is 0.800, average precision reached 0.996 for PCG-MambaConformer, whereas two models fell below the no-skill baseline (EfficientNet-B0 0.783 and 2D-CNN 0.686), consistent with their near-chance and sub-chance AUROC on that target.

The threshold comparison separated two failure modes that a single operating point conflates. On the heterogeneous target, recalibration changed very little: even with the unattainable target-optimal threshold, the best balanced accuracy was 0.682 (MaxViT), 0.679 (EfficientNet-B0) and 0.674 (CNN-BiLSTM), against 0.620, 0.662 and 0.640, respectively, at the fixed threshold, and the mean gain from optimal recalibration across the nine models was 0.022 (range 0.005–0.062). The failure on CinC is therefore one of discrimination rather than of threshold placement and cannot be repaired by recalibration.

On the homogeneous target the situation was the opposite. Several models whose behavior at the 0.5 threshold appeared to be a complete collapse in fact retained the information needed to separate the classes: under the target-optimal threshold, Hybrid-MLP moved from a balanced accuracy of 0.502 (sensitivity 0.004) to 0.902 (sensitivity 0.819, specificity 0.985), and Hybrid-KAN from 0.503 to 0.906 (sensitivity 0.833, specificity 0.980). The apparent collapse of these models on Yaseen was thus predominantly a threshold-transfer failure, which is precisely the discrepancy that a threshold-independent metric is designed to expose, and it accounts for the coexistence of high AUROC with near-zero sensitivity described in Section 3.1.

The practically relevant question for zero-shot use, however, is whether a decision threshold can be transferred without target labels. For most models it could not. Using the source-derived Youden threshold—the only rule available in a genuine zero-shot deployment—Hybrid-MLP remained at a balanced accuracy of 0.502 on Yaseen and Hybrid-KAN reached only 0.514, while EfficientNet-B0 and MaxViT moved further toward an always-abnormal decision (specificity 0.000 and 0.015, respectively). The largest improvement among the remaining models was for 1D-CNN (balanced accuracy 0.654 to 0.826), but with a sensitivity of 0.652 and a specificity of 1.000 its operating point remained below the balanced criterion. The single exception was PCG-MambaConformer, for which the source-derived threshold did transfer: balanced accuracy rose from 0.856 at the fixed threshold to 0.922 (sensitivity 0.863, specificity 0.980), approaching the 0.967 attainable only with target labels. Of the nine models, it was the only one for which a source-calibrated decision rule produced a balanced operating point on a previously unseen dataset without target labels; this is a technical property of threshold transfer on a curated benchmark and does not indicate clinical adequacy.

### 3.5. Domain Adaptation Is Conditional

**Figure 3:**
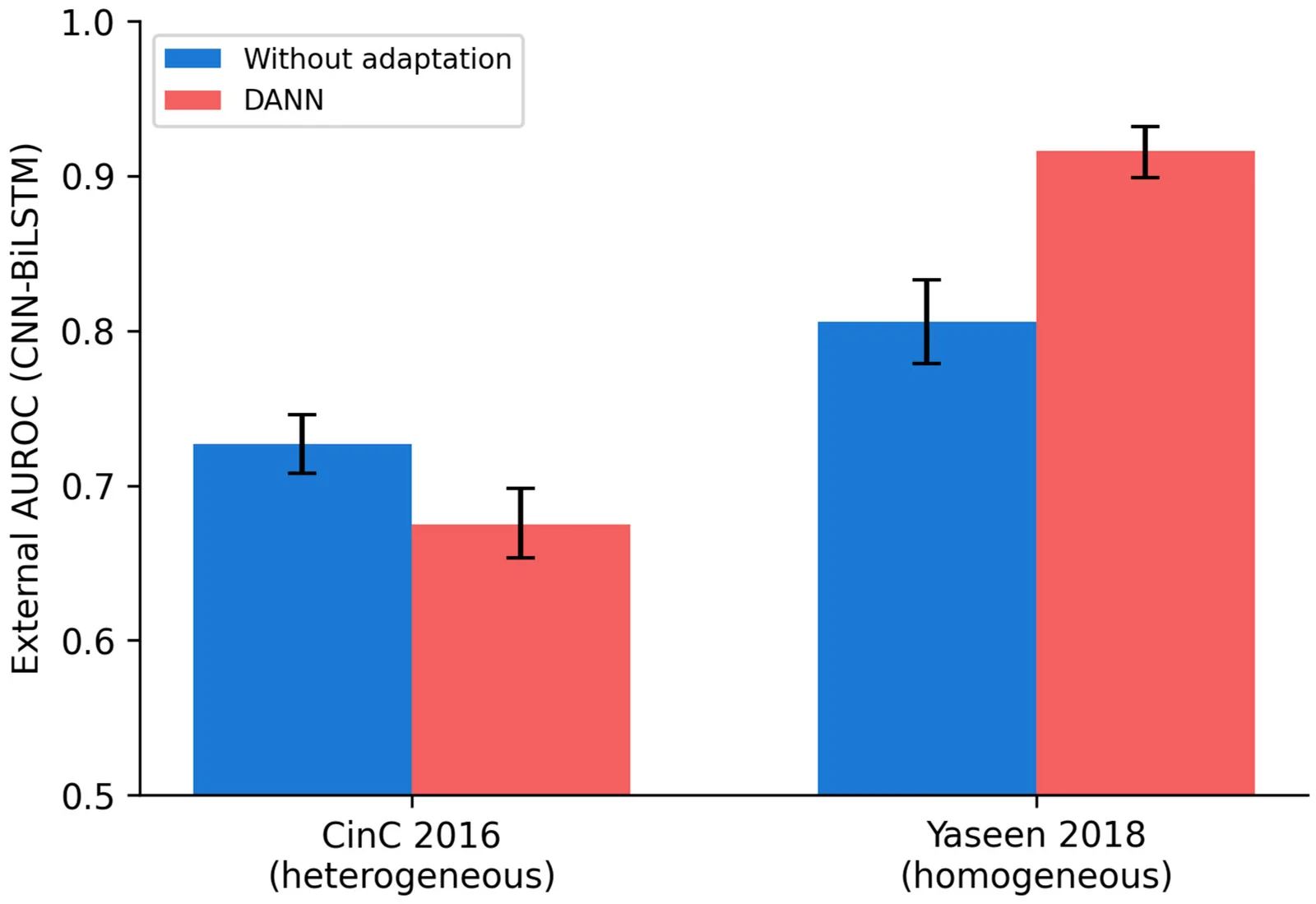
Effect of adversarial domain adaptation (DANN) on cross-dataset AUROC for the CNN-BiLSTM model, shown separately for the homogeneous (Yaseen 2018 [29]) and heterogeneous (CinC 2016 [5,6]) targets. Bars show AUROC with 95% bootstrap confidence intervals (2000 resamples). DANN increased AUROC on Yaseen 2018 and decreased it on CinC 2016; both differences were significant (DeLong test comparing the non-adapted and DANN models on the same target, *p* < 0.001 for each). The vertical axis starts at 0.5 (chance level).

Adversarial domain adaptation (DANN) applied to the CNN-BiLSTM model had opposite effects on the two external datasets. On the homogeneous Yaseen target, adaptation increased AUROC from 0.806 to 0.916 (DeLong *p* < 0.001). On the heterogeneous CinC target, adaptation decreased AUROC from 0.727 to 0.675 (DeLong *p* < 0.001). Figure 3 illustrates this conditional effect. The result indicates that adversarial alignment to an unlabeled target was beneficial only when that target was internally consistent; for a multi-source target, alignment toward a heterogeneous mixture reduced performance.

### 3.6. Disease-Specific Transfer, Leakage Check, and Label-Grouping Sensitivity Analysis

**Figure 4:**
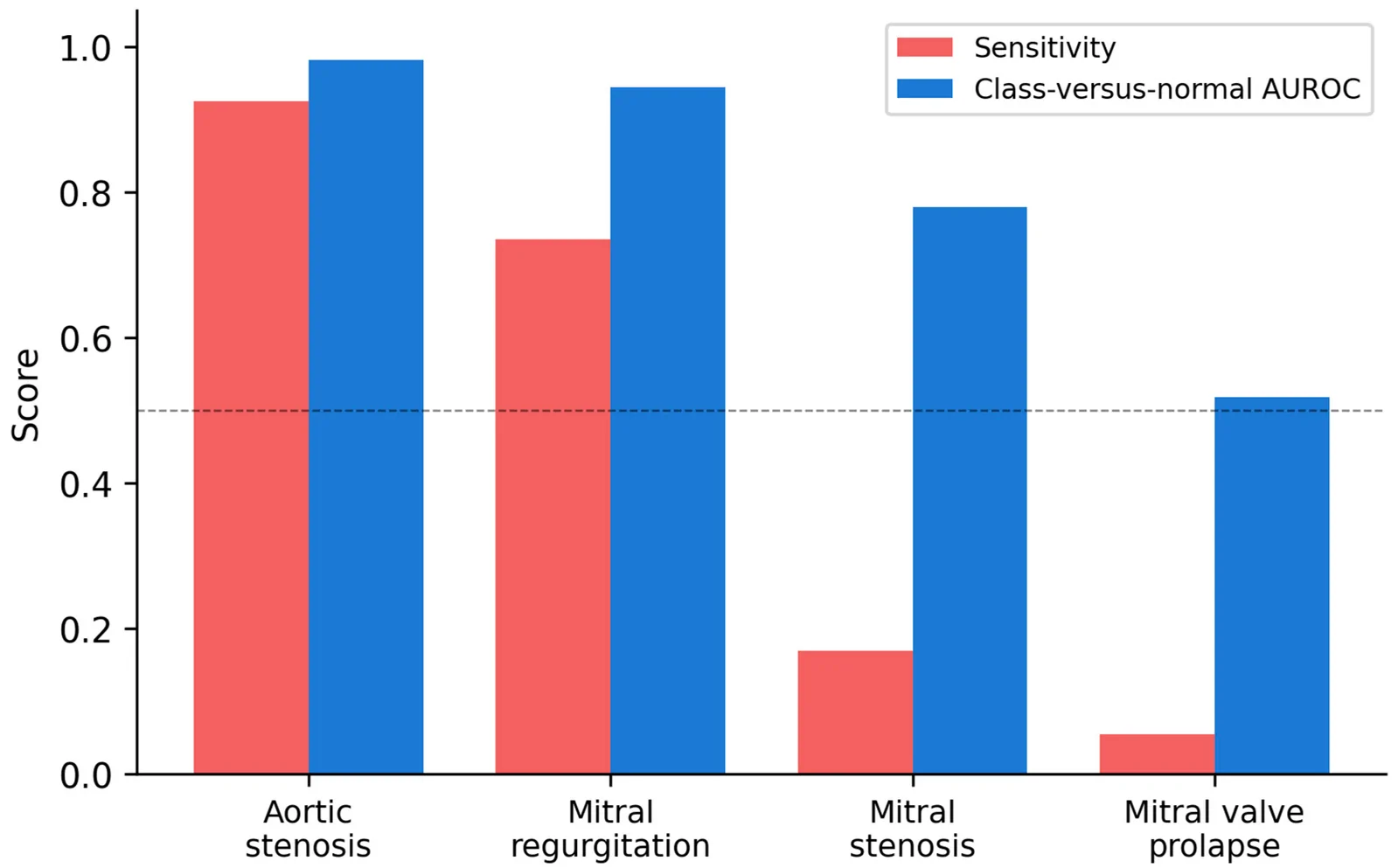
Disease-specific cross-dataset transfer (CirCor to Yaseen) for the CNN-BiLSTM model, showing per-class sensitivity and class-versus-normal AUROC; the horizontal dashed line marks chance level (0.5).

A disease-specific analysis clarified why transfer to the homogeneous target was uneven across pathologies. For the CNN-BiLSTM model evaluated per Yaseen class, aortic stenosis and mitral regurgitation transferred well (sensitivity 0.925 and 0.735; class-versus-normal AUROC 0.982 and 0.945), whereas mitral stenosis and mitral valve prolapse transferred poorly (sensitivity 0.170 and 0.055; class-versus-normal AUROC 0.780 and 0.518). This pattern is consistent with the acoustic prominence of the murmurs: loud, higher-frequency murmurs were detected across datasets, whereas softer or click-dominated findings were not. Figure 4 shows the per-class transfer results.

To exclude data leakage as an explanation for the high Yaseen performance, record identifiers were compared between the internal and external datasets; no overlapping records were found, confirming that external evaluation was zero-shot. The CWT representation was confirmed to differ from the mel representation (correlation 0.44), indicating that it contributed distinct information rather than duplicating the spectrogram.

A sensitivity analysis examined whether grouping the 131 unknown-murmur recordings with the abnormal class influenced the findings. Two representative models—CNN-BiLSTM and PCG-MambaConformer—were retrained after excluding the unknown recordings (Present-versus-Absent only) and re-evaluated on both external datasets (Supplementary Table S2). A consistent pattern emerged: excluding the unknown class raised internal AUROC (CNN-BiLSTM 0.903 to 0.965; PCG-MambaConformer 0.862 to 0.948) but lowered performance on both external targets (PCG-MambaConformer Yaseen 0.985 to 0.893 and CinC 0.601 to 0.447; CNN-BiLSTM Yaseen 0.806 to 0.483 and CinC 0.727 to 0.662). The grouping therefore did influence the absolute external values rather than being inconsequential; however, the direction of this effect reinforces the central finding of the study, because removing the ambiguous cases improved the internal estimate while degrading external transfer. Discrimination by PCG-MambaConformer on the homogeneous target remained high without the unknown recordings (Yaseen AUROC 0.893; operating points in Supplementary Table S2), but the reported operating points should be read as conditional on the conservative grouping in which unknown murmurs are treated as abnormal.

### 3.7. Uncertainty, Ensembling, and Ablation

**Figure 5:**
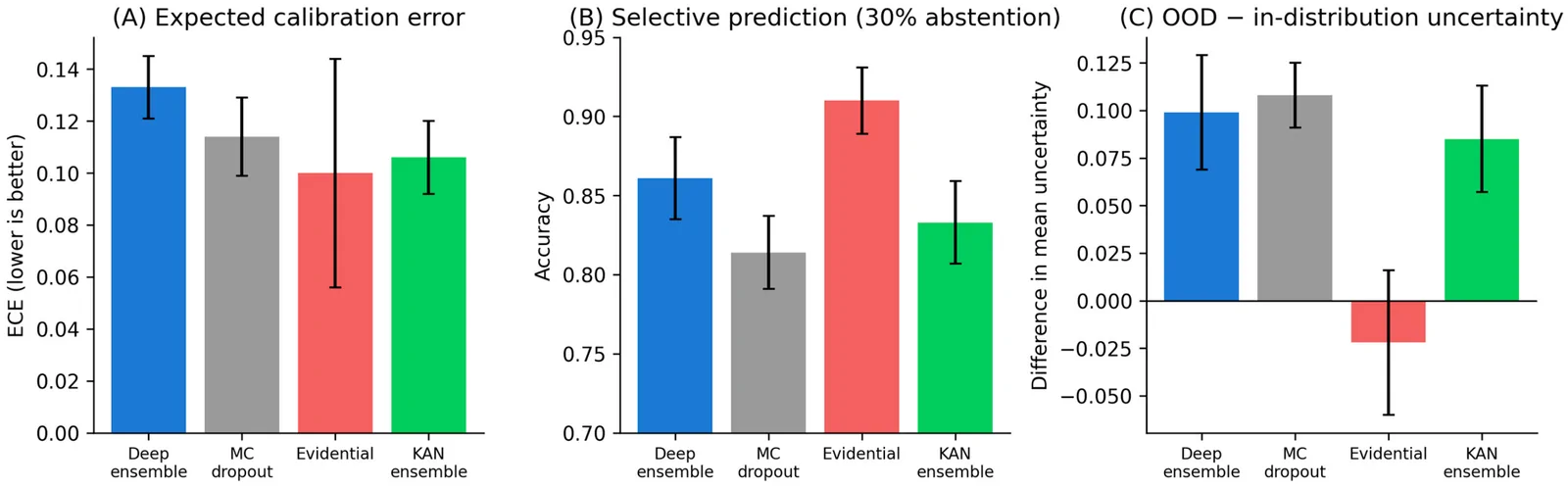
Uncertainty-quantification benchmark across five folds for four methods (deep ensemble, Monte Carlo dropout, evidential, and Kolmogorov–Arnold network ensemble), showing (**A**) the expected calibration error, (**B**) selective-prediction accuracy and (**C**) the difference in mean uncertainty between out-of-distribution and in-distribution inputs. Bars show the mean ± standard deviation across the five folds. Selective-prediction accuracy is computed after abstaining on the 30% most uncertain cases; in panel (**C**), out-of-distribution inputs are PhysioNet/CinC 2016 [5,6] recordings and in-distribution inputs are CirCor [7] recordings; a negative value indicates lower uncertainty on out-of-distribution inputs. Because the evidential head uses a different uncertainty measure (vacuity) from the other methods (predictive entropy), the sign rather than the magnitude of the difference is comparable across methods.

The uncertainty-quantification benchmark showed that the four methods were complementary rather than uniformly ranked. Ensemble- and dropout-based methods produced positive separation between in-distribution and out-of-distribution uncertainty, whereas the evidential method achieved the strongest selective-prediction performance but a slightly negative mean in-/out-of-distribution uncertainty difference whose fold-to-fold variation included zero, indicating that it did not reliably flag out-of-distribution inputs. Figure 5 summarizes the calibration and selective-prediction results across the five folds. This behavior is relevant to the safety of any future deployment because a method that abstains well in-distribution may still fail to flag genuinely novel recordings.

**Figure 6:**
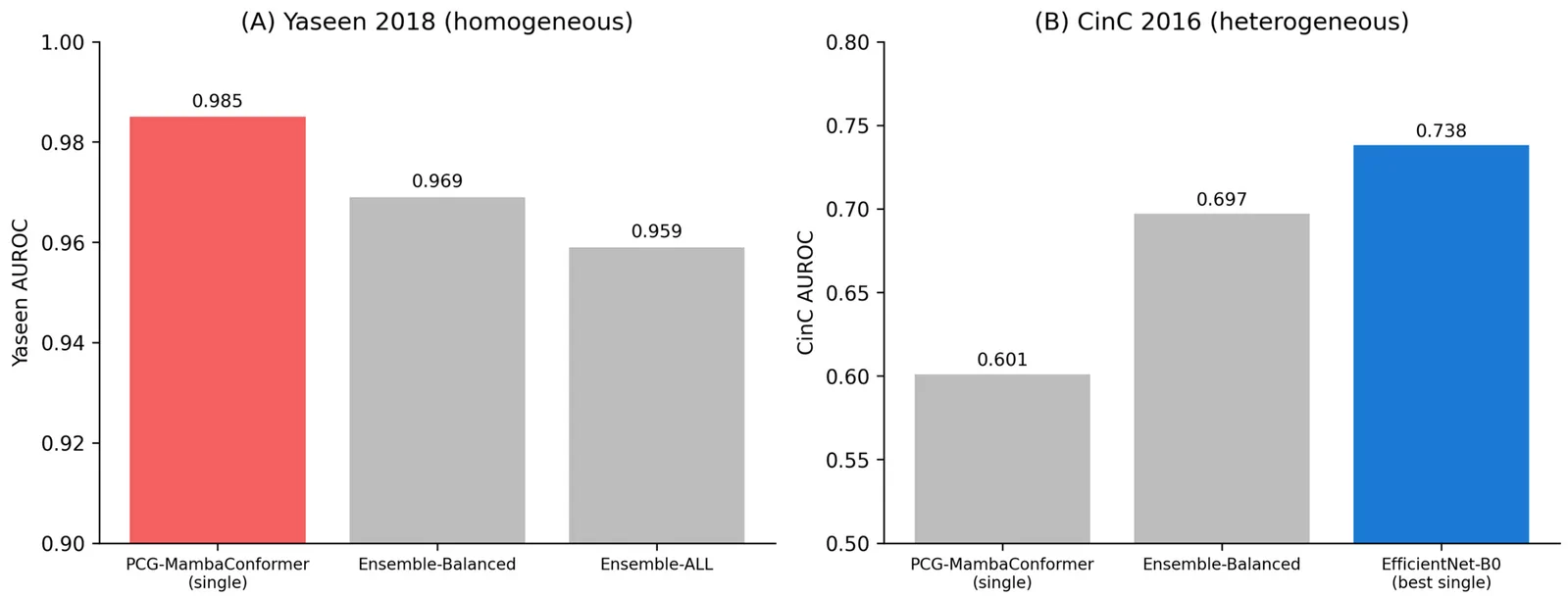
Multi-architecture ensembling compared to single models on the homogeneous (Yaseen 2018 [29], panel (**A**)) and heterogeneous (CinC 2016 [5,6], panel (**B**)) targets. Ensemble-ALL denotes soft voting over all nine deep models and Ensemble-Balanced soft voting over the two-model subset (CNN-BiLSTM and PCG-MambaConformer). Values above the bars are AUROC; the best-performing configuration on each target is shown in color and the remaining configurations in grey.

Multi-architecture ensembling did not resolve the cross-dataset problem. On the homogeneous target, the single best model (PCG-MambaConformer, AUROC 0.985) exceeded both a naive all-model ensemble (0.959) and Ensemble-Balanced (0.969); adding weaker models reduced the ensemble sensitivity relative to the single model (0.510 for Ensemble-ALL and 0.573 for Ensemble-Balanced versus 0.718, all at a specificity of at least 0.995). On the heterogeneous target, ensembling produced an increase over the proposed model (Ensemble-Balanced, 0.697 versus 0.601) but remained below the best single model (EfficientNet-B0, 0.738) and discrimination remained modest. Figure 6 shows the ensembling results for both datasets.

**Figure 7:**
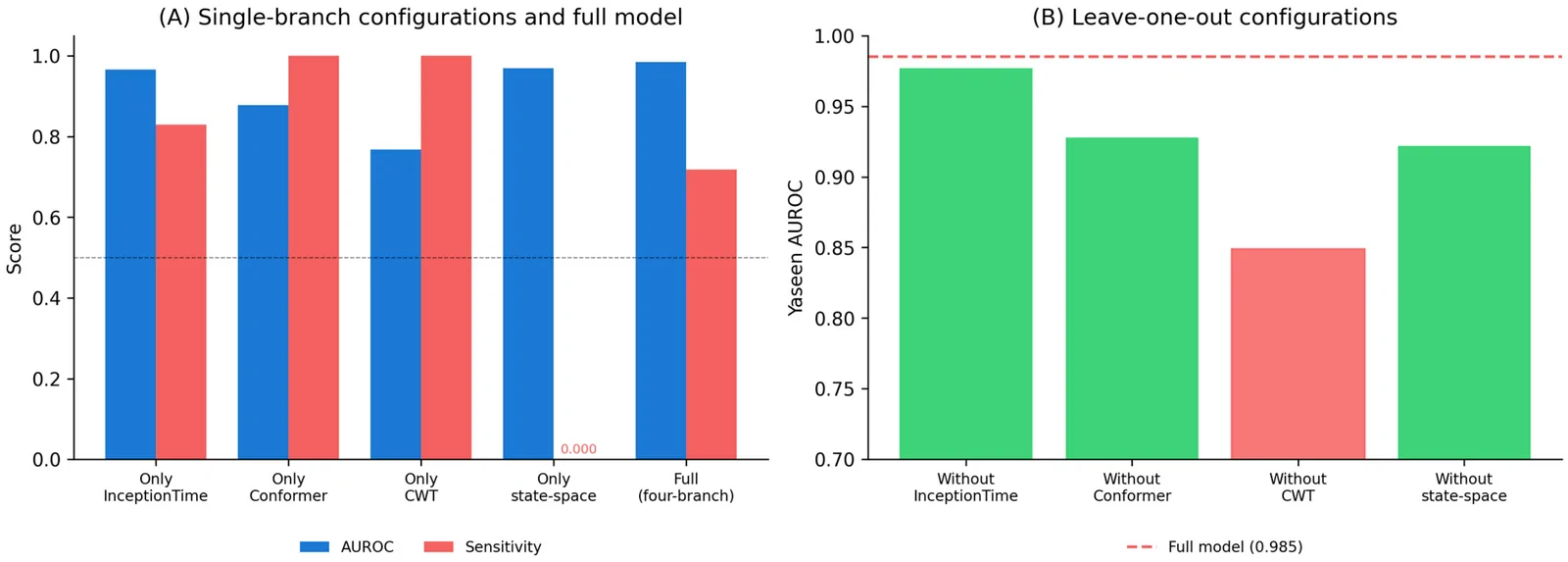
Branch ablation of PCG-MambaConformer: (**A**) single-branch configurations and the full model, showing AUROC and sensitivity; (**B**) leave-one-out configurations, showing the AUROC decrease relative to the full model. The dotted horizontal line in (A) marks 0.5.

The branch ablation indicated that the cross-dataset behavior of the proposed model depended largely on the combination of branches. As shown in Table 4 and Figure 7, three of the four single-branch configurations degenerated toward an extreme operating point even when their AUROC was high: the Conformer and CWT branches alone classified all recordings as abnormal (sensitivity 1.000, specificity 0.000; AUROC 0.878 and 0.768), whereas the state-space branch alone classified all recordings as normal (sensitivity 0.000, specificity 1.000; AUROC 0.969). The raw-waveform InceptionTime branch was the exception, retaining a balanced operating point (sensitivity 0.830, specificity 0.980) with a lower AUROC than the full model (0.966 versus 0.985). Among leave-one-out configurations, removing the CWT branch produced the largest decrease in AUROC (from 0.985 to 0.849), identifying CWT as an important but not self-sufficient component. The full four-branch model achieved the highest AUROC of all configurations, with a sensitivity of 0.718 and a specificity of 0.995.

### 3.8. Feature-Space and Saliency Analysis

**Figure 8:**
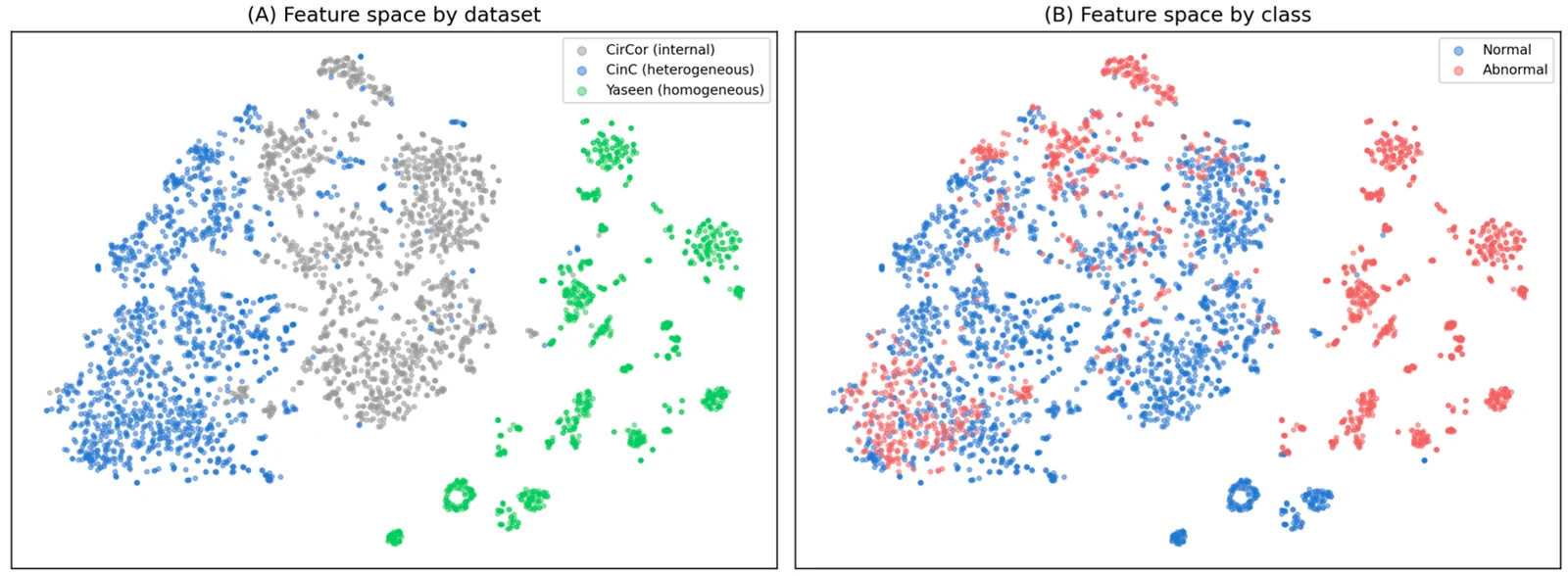
Two-dimensional t-SNE projection of the pre-fusion features of PCG-MambaConformer for all three datasets: (**A**) colored by dataset; (**B**) colored by class (normal versus abnormal). The homogeneous Yaseen recordings form compact, class-pure clusters, whereas the heterogeneous CinC recordings overlap with the internal distribution and show mixed classes.

To examine why transfer succeeded on the homogeneous target but failed on the heterogeneous one, the pre-fusion features of PCG-MambaConformer were projected to two dimensions with t-distributed stochastic neighbor embedding (t-SNE) for all three datasets. Figure 8 shows the resulting feature space, colored by dataset (panel A) and by class (panel B). In panel A, the homogeneous Yaseen recordings formed compact, isolated clusters, whereas the heterogeneous CinC recordings were intermixed with the internal CirCor distribution. In panel B, the Yaseen clusters were class-pure (each cluster predominantly normal or abnormal), while the CinC region showed substantial overlap between normal and abnormal recordings.

Importantly, the simple notion of domain proximity did not account for transfer: the t-SNE centroid distance from CirCor was larger for Yaseen than for CinC, even though transfer was far better for Yaseen. A class-separability analysis in the original 512-dimensional feature space was more informative. The silhouette score for the normal-versus-abnormal partition was highest for Yaseen (0.288), intermediate for the internal CirCor data (0.164), and lowest for CinC (0.032); the corresponding linear-separability AUROC (five-fold logistic regression) was 1.000 for Yaseen, 0.750 for CirCor, and 0.925 for CinC. The high silhouette score for Yaseen indicates that its classes formed compact, well-separated clusters in the learned representation, consistent with its strong zero-shot performance, whereas the low silhouette score for CinC reflects a diffuse, overlapping class structure. The apparently high CinC linear-separability AUROC (0.925) is not inconsistent with this and merits clarification: the silhouette score measures the compactness and Euclidean isolation of the class clusters, whereas the linear-separability AUROC measures only whether a separating hyperplane exists, and it was obtained by fitting a logistic-regression classifier to each dataset’s own labels. The CinC classes are thus linearly separable in-domain (0.925) without forming compact clusters (silhouette 0.032). This in-domain separability is not what zero-shot transfer uses: the low external AUROC for CinC (0.601) shows that the decision boundary learned on CirCor does not align with the CinC class structure. The discriminative information is therefore present in the representation, but the source-trained boundary is positioned incorrectly for the heterogeneous target—a boundary-transfer failure under distribution shift rather than an absence of class information. Of the metrics examined, the silhouette score (cluster compactness) tracked transfer quality across the three datasets, whereas in-domain linear separability did not. In the datasets evaluated here, cross-dataset transfer was therefore associated with the compactness and separability of the target classes in the learned feature space rather than with the proximity of dataset centroids.

**Figure 9:**
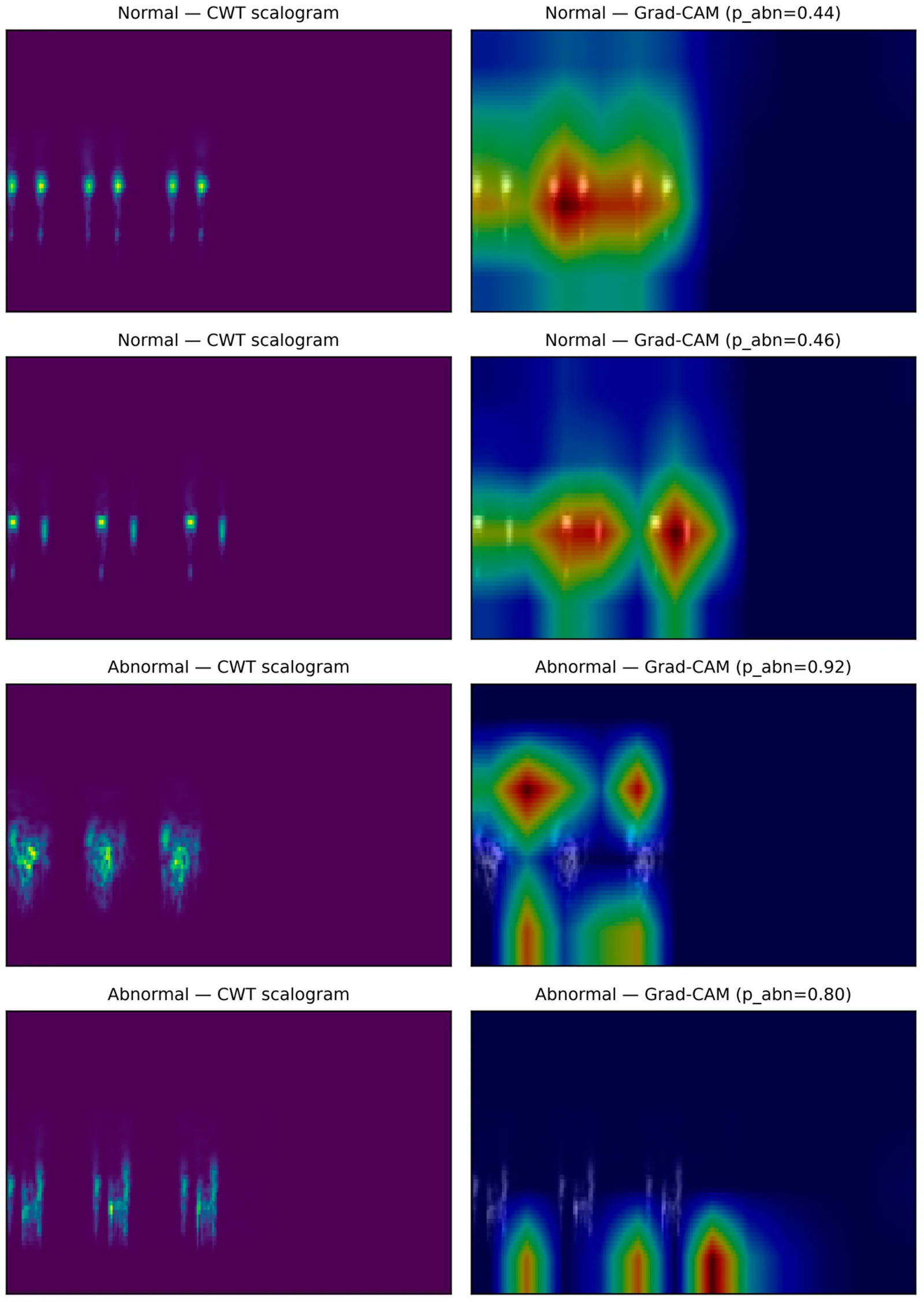
Grad-CAM saliency on the CWT branch of PCG-MambaConformer for correctly classified Yaseen recordings (two normal, two abnormal). (**Left**): CWT scalogram; (**right**): Grad-CAM overlay, with the predicted probability of the abnormal class (p_abn) indicated above each panel. In the scalograms, color encodes the log-magnitude of the Morlet CWT coefficients, standardized within each segment (brighter = higher magnitude). In the Grad-CAM overlays, the saliency map is superimposed on the grayscale scalogram and min–max normalized within each recording; warmer colors (red) indicate time–scale regions that contributed more strongly to the predicted class, and cooler colors (blue) indicate weaker contribution.

To examine which parts of the input the model relied on, Grad-CAM was computed on the CWT branch for correctly classified Yaseen recordings. Figure 9 shows representative normal and abnormal examples with their scalograms and the corresponding saliency maps. The salient regions concentrated on the periodic high-energy time–frequency bands corresponding to the principal heart-sound events rather than on background regions, and the predicted abnormal probabilities fell on the correct side of the 0.5 threshold (0.44 and 0.46 for the normal examples, close to the threshold, and 0.80 and 0.92 for the abnormal examples). This indicates that the model based its decisions on physiologically plausible acoustic structure, supporting the interpretability of the representation underlying the cross-dataset results.

## 4. Discussion

This study evaluated cross-dataset generalization in PCG murmur classification across eleven approaches and two external datasets. The central observation is that, in the two external datasets evaluated, successful generalization was associated with the homogeneity of the target dataset. These two targets differ from one another in more than homogeneity alone—population, recording protocol, disease spectrum, class distribution and signal quality all differ—so this association should be read as a plausible and testable correlate of transferability in the present comparison rather than as an established general determinant. On a homogeneous, single-protocol target, a multi-representation fusion model achieved balanced and reproducible performance (AUROC 0.985), whereas on a heterogeneous, multi-source target no method—classical, deep, image-based, adapted, or ensembled—achieved more than modest discrimination (highest AUROC 0.738). These two findings are complementary rather than contradictory: the same model that transferred well to a consistent target failed on an inconsistent one.

A practical implication concerns evaluation. Internal AUROC was a weak predictor of external behavior, and several models with high internal and external AUROC nonetheless operated at degenerate (one-class) operating points after transfer, with sensitivity or specificity close to 0. This reinforces the recommendation, made in the broader clinical-prediction literature, that internal cross-validation is insufficient evidence of generalizability and that external evaluation with multiple operating-point metrics is necessary [22,24,41]. The contrast between AUROC and the sensitivity/specificity balance was particularly informative: a single threshold-independent metric concealed model collapse that became obvious only when the operating point was examined.

The threshold analysis refines this point by separating two failure modes that a single operating point conflates. On the heterogeneous target, an oracle threshold chosen with full knowledge of the target labels raised balanced accuracy by only 0.022 on average, so the failure there is one of discrimination: no recalibration would yield adequate performance, and different data or a different modeling strategy would be required. On the homogeneous target the opposite applied, with models that appeared to have collapsed entirely at the 0.5 threshold recovering to balanced accuracies above 0.90 once the cut-off was chosen on the target, meaning the discriminative information was present and only the decision boundary was misplaced. The distinction is practically consequential because the two situations call for different remedies, and it also qualifies how Table 2 should be read: the collapsed operating points on the homogeneous target are evidence that a source-calibrated threshold does not transfer, not evidence that the models learned nothing. The practical caveat is that a threshold derived from the source domain—the only rule available without target labels—failed to resolve the imbalance for eight of the nine models, and only the multi-representation fusion model produced a balanced operating point on an unseen dataset without any target supervision. Threshold recalibration is therefore necessary but not sufficient, and obtaining even a small labeled calibration sample from the intended deployment site would be a prerequisite before any deployment could be considered.

The conditional effect of adversarial domain adaptation is consistent with the assumptions underlying such methods. Domain-adversarial training aligns source and target feature distributions; when the target is internally consistent, this alignment is well-defined and beneficial, but when the target is a heterogeneous mixture, aligning to its aggregate distribution can be counterproductive. This observation cautions against applying unsupervised adaptation uniformly without first characterizing the target. The disease-specific analysis provides a mechanistic complement: pathologies with acoustically prominent murmurs transferred across datasets, whereas softer or click-dominated findings did not, which helps explain both the success on a homogeneous valvular dataset and the difficulty on heterogeneous recordings.

The ablation results bear on the interpretation of the proposed model. Rather than a single representation accounting for performance, the balanced behavior emerged from the combination of branches: three of the four branches individually collapsed toward predicting one class, the raw-waveform InceptionTime branch being the exception, and their fusion produced the highest AUROC together with a stable operating point. The continuous wavelet representation was the most influential single component, consistent with prior work using wavelet-based time–frequency images for heart-sound classification [15], but it was not sufficient on its own. This argues that the contribution of multi-representation fusion in this setting was to stabilize the operating point rather than to maximize a threshold-independent metric.

Why, then, did multi-representation fusion transfer so well to the homogeneous target yet fall behind simpler backbones on the heterogeneous one? Three differences between the two targets are relevant. First, the outcome label does not carry the same meaning in the two datasets. The Yaseen classes are defined by valvular lesions that by construction produce murmurs, so a decision function learned from murmur annotations is directly transferable. In PhysioNet/CinC 2016 the abnormal class was defined by a confirmed cardiac diagnosis and includes conditions such as coronary artery disease that frequently produce no murmur at all [5,6]; a murmur detector is therefore being asked to detect a partly inaudible outcome, which imposes a ceiling on achievable discrimination that no architecture can remove. This is label shift rather than covariate shift, and it is not addressed by feature alignment. Second, the four branches of the proposed model are all computed from the same band-limited, per-recording normalized waveform. When the target contributes little nuisance variation, the branches make partly independent errors and their fusion stabilizes the operating point, which is precisely the behavior observed in the ablation. Under multi-source shift, however, the dominant pre-analytical factors—sensor frequency response, ambient noise floor, auscultation position and recording length—perturb all four representations in the same direction, so branch errors become correlated, fusion no longer confers robustness, and the additional capacity is instead available to fit source-specific spectral detail. The heterogeneity of CinC 2016 is substantial in exactly these respects: its recordings range from 5.3 to 122.0 s (median 20.8) and are unevenly distributed across six sub-databases, two of which contribute 81% of the recordings. Third, the single-representation mel-spectrogram backbones (EfficientNet-B0, MaxViT) operate on a heavily smoothed representation whose coarse spectral envelope survives device and environment changes, which is consistent with their comparatively higher, though still modest, CinC AUROC. The heterogeneous result should therefore be read as the joint consequence of label shift, population shift and correlated pre-analytical corruption rather than as a shortcoming of representation fusion as such.

A distinct and clinically important component of the shift studied here is age. The internal dataset (CirCor DigiScope) was collected in a pediatric screening program, whereas PhysioNet/CinC 2016 is predominantly adult and multi-source and Yaseen 2018 is a valvular benchmark compiled from educational and online sources for which age information is not reported. Every external evaluation in the forward direction therefore involves transfer away from the pediatric source population. Heart rate, chest-wall acoustics and the relative frequency content of normal and pathological heart sounds differ with age, and the disease spectrum differs correspondingly: innocent flow murmurs and congenital lesions predominate in children, whereas degenerative valvular disease and ischemic heart disease predominate in adults. The poor performance on CinC may therefore reflect clinically meaningful population heterogeneity in addition to technical dataset heterogeneity. The disease-specific analysis is consistent with this reading, in that the pathologies which transferred best to the valvular target were those whose acoustic signature is least age-dependent (aortic stenosis, mitral regurgitation), whereas softer or click-dominated findings did not transfer. Two practical implications follow: a model intended for adult screening should not be developed on pediatric data alone, and the present results should not be read as an estimate of pediatric screening performance in a second pediatric cohort, which was not available here.

The reciprocal experiment allows the age-direction and the dataset-heterogeneity explanations to be partly separated. If the difficulty were primarily a consequence of developing the model on pediatric recordings, the reverse direction should have been easier; it was not. Training on PhysioNet/CinC 2016 and testing on CirCor yielded AUROC values of 0.677 (95% CI 0.633–0.718) for CNN-BiLSTM and 0.612 (0.565–0.657) for PCG-MambaConformer, in the same range as the 0.727 and 0.601 obtained in the forward direction, whereas the same CinC-trained models transferred well to the homogeneous Yaseen target (0.926 and 0.942). The barrier is therefore approximately symmetric and is attached to the heterogeneous dataset itself—as a source as well as a target—rather than to the pediatric origin of the training data alone. The age shift remains a genuine and clinically meaningful component of the problem, but it is not a sufficient explanation for the ceiling observed on CinC. The reciprocal results also reproduce the operating-point finding independently: trained on a different source, CNN-BiLSTM again collapsed on Yaseen (specificity 0.075) while the four-branch fusion model again retained a balanced operating point (sensitivity 0.894, specificity 0.875), which supports the interpretation that fusion stabilizes the decision boundary rather than merely improving a threshold-independent metric.

The label-grouping sensitivity analysis points in the same direction. Recordings annotated as unknown are by definition those in which the annotator could not confidently exclude a murmur, and they are consequently enriched for low-quality and acoustically ambiguous signals. Removing them produced a cleaner and easier internal task—internal AUROC rose by 0.062 for CNN-BiLSTM and by 0.086 for PCG-MambaConformer—yet degraded transfer to both external targets, with a marked loss on the heterogeneous CinC target for both models (0.727 to 0.662 and 0.601 to 0.447) and, for CNN-BiLSTM, a collapse on the homogeneous target (0.806 to 0.483). Exposure to ambiguous, lower-quality recordings during training therefore appears to act as an implicit robustness constraint that is most valuable precisely where the target is noisy, and its removal improves the internal estimate while making the model more brittle under shift. This is a further, independent illustration of the study’s central point that internal accuracy and external transfer can move in opposite directions.

The uncertainty analysis has a direct safety implication. The evidential method offered the best selective prediction in-distribution yet showed a slightly negative mean difference between in- and out-of-distribution uncertainty, meaning that it did not reliably assign higher uncertainty to genuinely novel inputs. Ensemble- and dropout-based methods were better at flagging out-of-distribution recordings. This mirrors reports in other domains that uncertainty estimates can remain useful under shift but are method-dependent and must be evaluated specifically for the out-of-distribution case [28]. For PCG screening, where novel devices and populations are expected, an uncertainty method that is confidently wrong on unfamiliar inputs would be hazardous.

The feature-space and saliency analyses provide a mechanistic reading of the transfer results. The t-SNE projection and the class-separability metrics indicated that what distinguished the easy (homogeneous) from the hard (heterogeneous) target was not the proximity of the target distribution to the training distribution, but the compactness and separability of the target classes in the learned representation. This reframes domain homogeneity in operational terms: a homogeneous target tends to occupy compact, class-pure regions that a source-trained model can separate, whereas a heterogeneous target spreads across overlapping regions. The Grad-CAM maps further indicated that, for correctly classified recordings, the model attended to the periodic high-energy components of the scalogram rather than to background, which is consistent with reliance on genuine heart-sound structure. Saliency methods such as Grad-CAM are increasingly used to make deep-learning decisions inspectable in clinical imaging [43], and here they support the interpretation that the representation, rather than a spurious cue, underlies the observed transfer behavior.

These findings should be read alongside the broader PCG literature, in which internal accuracies above 0.95 are frequently reported [10,16,17,44]. The present results do not contradict those reports but place them in context: high internal accuracy is achievable and was reproduced here, yet it did not guarantee transfer to an independent dataset, particularly a heterogeneous one. The comparatively modest external ceiling on the heterogeneous dataset is consistent with the difficulty of multi-source generalization observed across clinical machine learning [22,23].

The homogeneous target used here (Yaseen 2018) is itself a curated, high-signal-to-noise collection assembled from a limited set of online and textbook sources, and is widely regarded as a comparatively easy benchmark on which many models exceed 0.95 accuracy. The balanced transfer observed on this target should therefore be read as technical cross-dataset transfer under favorable, high-signal-to-noise conditions rather than as clinical external validation, not least because the operating point of the same model corresponds to a sensitivity of 0.718, so that roughly one in four abnormal recordings would still be missed at the 0.5 threshold. The CinC result, where the best AUROC across all eleven approaches was 0.738 and none of the nine deep models reached a sensitivity and a specificity both of at least 0.80 at any of the three decision thresholds examined, is the more realistic indication of what current PCG classifiers achieve outside their development domain.

This study has several limitations. Model development used a single internal dataset (pediatric CirCor). To test whether the observed difficulty was a property of that choice, a reciprocal experiment was added in which the same two representative models were retrained on PhysioNet/CinC 2016 and evaluated on CirCor; the results are reported in Supplementary Table S8 and discussed above. Training on more than two sources, and prospective collection in the intended deployment setting, remain outside the scope of the present datasets. The binary normal-versus-abnormal framing, although necessary for cross-dataset comparability, discards finer murmur categories present in the annotations; moreover, the sensitivity analysis showed that the choice to group unknown-murmur recordings with the abnormal class measurably affected the absolute external values, so the reported figures are conditional on this conservative labeling decision. The two external datasets, while contrasting, do not span the full range of devices and populations encountered clinically; as noted in the first paragraph of the Discussion, the association between target homogeneity and transfer rests on only two external targets and remains a hypothesis. Operating points were examined at three cut-offs—the fixed 0.5 threshold, a threshold derived from the source domain by Youden’s J, and an oracle threshold optimized on the target itself—but the intermediate and practically relevant case of calibrating on a small labeled sample from the target site was not explored; such calibration requires target labels that are, by definition, unavailable in the zero-shot setting evaluated here, and it would mitigate a threshold-transfer failure without repairing a discrimination failure. Finally, the proposed model was evaluated as a research prototype and was not tested prospectively or in a clinical workflow.

Future work could examine multi-source training to improve heterogeneous transfer, target-aware adaptation that first assesses target homogeneity, calibration of operating points on limited labeled target data, and prospective evaluation on recordings collected with the devices and in the settings intended for deployment.

## 5. Conclusions

In the two external datasets evaluated in this study, successful cross-dataset generalization in PCG murmur classification was associated with the homogeneity of the target dataset. Because these datasets also differed in population, recording protocol, disease spectrum, class distribution and signal quality, target homogeneity should be regarded as a plausible correlate of transferability in this comparison rather than as an established general determinant, and it requires confirmation across a larger and more diverse set of datasets. Internal accuracy was a weak indicator of external performance, and model complexity alone did not close the transfer gap to a heterogeneous, multi-source dataset. On a homogeneous target, balanced and reproducible generalization was achieved by a multi-representation fusion model, and an ablation indicated that most single representations collapsed to a one-class operating point, whereas their fusion achieved the highest AUROC. Adversarial domain adaptation helped only when the target was internally consistent, and uncertainty methods varied in their reliability on out-of-distribution inputs. The balanced performance achieved on the homogeneous target should be interpreted as evidence of technical cross-dataset transfer under favorable, highly curated conditions rather than as clinical external validation; the heterogeneous target, where the best AUROC was 0.738, is the more realistic indication of present capability. Together, these results argue for multi-metric evaluation on independent external data, followed by prospective clinical validation, and for matching the modeling and adaptation strategy to the characteristics of the intended target domain before any clinical use is considered.

## Figures and Tables

**Table 1:** Internal and external AUROC with 95% confidence intervals for the nine deep models (internal: patient level; external: recording level; internal AUROC on pooled out-of-fold cross-validation predictions, external AUROC on the five-fold ensemble). Confidence intervals were obtained by stratified bootstrap (2000 resamples). Abbreviations: AUROC, area under the receiver operating characteristic curve; CinC, the PhysioNet/CinC 2016 dataset (heterogeneous external target); Yaseen, the Yaseen 2018 dataset (homogeneous external target).

Approach	Internal AUROC (95% CI)	CinC AUROC (95% CI)	Yaseen AUROC (95% CI)
1D-CNN	0.832 (0.799–0.863)	0.513 (0.488–0.536)	0.891 (0.871–0.910)
2D-CNN	0.830 (0.795–0.861)	0.667 (0.645–0.689)	0.255 (0.215–0.297)
ResNet1D	0.823 (0.788–0.857)	0.579 (0.555–0.602)	0.852 (0.828–0.874)
CNN-BiLSTM	0.903 (0.876–0.927)	0.727 (0.708–0.746)	0.806 (0.779–0.833)
Hybrid-MLP	0.889 (0.858–0.916)	0.508 (0.484–0.532)	0.882 (0.862–0.902)
Hybrid-KAN	0.900 (0.871–0.925)	0.571 (0.546–0.595)	0.909 (0.891–0.926)
EfficientNet-B0	0.862 (0.827–0.893)	0.738 (0.719–0.758)	0.485 (0.439–0.534)
MaxViT	0.865 (0.832–0.895)	0.729 (0.709–0.749)	0.821 (0.793–0.846)
PCG-MambaConformer	0.862 (0.829–0.893)	0.601 (0.576–0.624)	0.985 (0.977–0.992)

Two classical baselines (SVM-RBF, random forest) are summarized in the text.

**Table 2:** Operating points (sensitivity and specificity at the 0.5 decision threshold) for the nine deep models, with 95% Wilson score confidence intervals. Internal performance is at the patient level (197 abnormal, 556 normal patients); external performance is at the recording level (CinC 2016 [5,6]: 665 abnormal, 2575 normal; Yaseen 2018 [29]: 800 abnormal, 200 normal). Sens = sensitivity; Spec = specificity.

Approach	Internal Sens/Spec (95% CI)	CinC Sens/Spec (95% CI)	Yaseen Sens/Spec (95% CI)
1D-CNN	0.594 (0.524–0.660)/0.847 (0.815–0.875)	0.063 (0.047–0.084)/0.982 (0.976–0.986)	0.308 (0.277–0.340)/1.000 (0.981–1.000)
2D-CNN	0.736 (0.670–0.793)/0.712 (0.673–0.748)	0.782 (0.749–0.812)/0.457 (0.438–0.476)	1.000 (0.995–1.000)/0.000 (0.000–0.019)
ResNet1D	0.594 (0.524–0.660)/0.887 (0.858–0.910)	0.126 (0.103–0.154)/0.942 (0.932–0.950)	0.324 (0.292–0.357)/1.000 (0.981–1.000)
CNN-BiLSTM	0.746 (0.681–0.802)/0.899 (0.872–0.922)	0.502 (0.464–0.540)/0.777 (0.761–0.793)	0.471 (0.437–0.506)/1.000 (0.981–1.000)
Hybrid-MLP	0.609 (0.540–0.675)/0.957 (0.937–0.971)	0.137 (0.113–0.165)/0.897 (0.885–0.908)	0.004 (0.001–0.011)/1.000 (0.981–1.000)
Hybrid-KAN	0.721 (0.654–0.779)/0.903 (0.875–0.925)	0.159 (0.134–0.189)/0.917 (0.906–0.927)	0.006 (0.003–0.015)/1.000 (0.981–1.000)
EfficientNet-B0	0.482 (0.414–0.552)/0.986 (0.972–0.993)	0.534 (0.496–0.571)/0.791 (0.775–0.806)	0.896 (0.873–0.916)/0.090 (0.058–0.138)
MaxViT	0.690 (0.623–0.751)/0.887 (0.858–0.910)	0.398 (0.362–0.436)/0.841 (0.827–0.855)	0.996 (0.989–0.999)/0.080 (0.050–0.126)
PCG-MambaConformer	0.650 (0.581–0.713)/0.915 (0.889–0.936)	0.198 (0.170–0.231)/0.932 (0.922–0.942)	0.718 (0.685–0.748)/0.995 (0.972–0.999)

A high AUROC frequently coexisted with a strongly imbalanced operating point after transfer; the narrowness of the intervals indicates that this is a reproducible property of the transferred decision boundary rather than sampling noise.

**Table 3:** Operating-point transfer on the two external datasets. AP = average precision (the no-skill baseline equals the abnormal prevalence of each dataset). Sensitivity and specificity are reported at three thresholds: the fixed 0.5 cut-off; a source-derived threshold obtained by maximizing Youden’s J statistic on the internal CirCor out-of-fold predictions (the only rule available without target labels); and the target-optimal threshold, an oracle that requires target labels and is therefore unattainable in a zero-shot setting. BA = balanced accuracy, (sensitivity + specificity)/2, given for the three thresholds in the same order. Precision–recall curves are shown in Supplementary Figure S1.

CinC 2016 [5,6] (Heterogeneous; Abnormal Prevalence 0.205)					
**Approach**	**AP**	**Sens/Spec @ 0.5**	**Sens/Spec @ Source J**	**Sens/Spec @ Oracle**	**BA (0.5/Source J/Oracle)**
1D-CNN	0.249	0.063/0.982	0.713/0.313	0.920/0.141	0.522/0.513/0.531
2D-CNN	0.358	0.782/0.457	0.600/0.635	0.779/0.470	0.619/0.618/0.624
ResNet1D	0.301	0.126/0.942	0.090/0.976	0.823/0.304	0.534/0.533/0.563
CNN-BiLSTM	0.410	0.502/0.777	0.490/0.787	0.722/0.626	0.640/0.639/0.674
Hybrid-MLP	0.243	0.137/0.897	0.189/0.817	0.931/0.142	0.517/0.503/0.536
Hybrid-KAN	0.294	0.159/0.917	0.214/0.856	0.728/0.383	0.538/0.535/0.555
EfficientNet-B0	0.412	0.534/0.791	0.755/0.573	0.695/0.663	0.662/0.664/0.679
MaxViT	0.390	0.398/0.841	0.612/0.707	0.788/0.576	0.620/0.660/0.682
PCG-MambaConformer	0.345	0.198/0.932	0.263/0.878	0.308/0.839	0.565/0.571/0.574
**Yaseen 2018 [29] (Homogeneous; Abnormal Prevalence 0.800)**					
**Approach**	**AP**	**Sens/Spec @ 0.5**	**Sens/Spec @ Source J**	**Sens/Spec @ Oracle**	**BA (0.5/Source J/Oracle)**
1D-CNN	0.974	0.308/1.000	0.652/1.000	0.761/0.955	0.654/0.826/0.858
2D-CNN	0.686	1.000/0.000	1.000/0.000	0.011/1.000	0.500/0.500/0.506
ResNet1D	0.965	0.324/1.000	0.131/1.000	0.698/0.980	0.662/0.566/0.839
CNN-BiLSTM	0.952	0.471/1.000	0.458/1.000	0.579/1.000	0.736/0.729/0.789
Hybrid-MLP	0.974	0.004/1.000	0.004/1.000	0.819/0.985	0.502/0.502/0.902
Hybrid-KAN	0.979	0.006/1.000	0.028/1.000	0.833/0.980	0.503/0.514/0.906
EfficientNet-B0	0.783	0.896/0.090	0.998/0.000	0.804/0.275	0.493/0.499/0.539
MaxViT	0.953	0.996/0.080	1.000/0.015	0.610/0.960	0.538/0.508/0.785
PCG-MambaConformer	0.996	0.718/0.995	0.863/0.980	0.954/0.980	0.856/0.922/0.967

**Table 4:** Branch ablation of PCG-MambaConformer on the homogeneous Yaseen dataset. Each configuration was retrained with five-fold cross-validation.

Configuration	Yaseen AUROC	Sensitivity	Specificity
Only InceptionTime branch	0.966	0.830	0.980
Only Conformer branch	0.878	1.000	0.000
Only CWT branch	0.768	1.000	0.000
Only state-space branch	0.969	0.000	1.000
Without InceptionTime branch	0.977	0.194	1.000
Without Conformer branch	0.928	0.305	1.000
Without CWT branch	0.849	0.695	0.810
Without state-space branch	0.922	0.644	1.000
Full (four-branch)	0.985	0.718	0.995

AUROC = area under the receiver operating characteristic curve; sensitivity and specificity are reported at a 0.5 decision threshold.

## Data Availability

The three datasets analyzed in this study are publicly available. The CirCor DigiScope dataset (https://physionet.org/content/circor-heart-sound/1.0.3/, accessed on 25 September 2026) and the PhysioNet/CinC Challenge 2016 dataset (https://physionet.org/content/challenge-2016/1.0.0/, accessed on 25 September 2026) are available from PhysioNet, and the Yaseen 2018 dataset is available from the repository accompanying its original publication [29] (https://github.com/yaseen21khan/Classification-of-Heart-Sound-Signal-Using-Multiple-Features-, accessed on 25 September 2026). The source code used for model development and cross-dataset evaluation is openly available at https://github.com/huseyinKutlu/PCG-CrossDataset (accessed on 25 September 2026) and archived on Zenodo (v1.0.1; https://doi.org/10.5281/zenodo.22957181).

## Supplementary Materials

The following supporting information can be downloaded at: https://www.mdpi.com/article/10.3390/jcm15208058/s1, Table S1: Common preprocessing and time–frequency representation parameters applied identically to all three datasets; Table S2: Training configuration shared by the deep models, and the additional settings for the domain-adaptation and uncertainty analyses; Table S3: Software library versions, hardware, and reproducibility settings; Table S4: Number of recordings and 5-second segments per dataset after preprocessing; Table S5: Effect of excluding the 131 unknown-murmur recordings from training; Table S6: TRIPOD+AI reporting map; Table S7: CLAIM (2024 update) reporting map; Table S8: Reciprocal (reverse-direction) cross-dataset transfer; Table S9: Operating-point analysis on the internal CirCor dataset (patient level; abnormal prevalence 0.262); Figure S1: Precision–recall curves for the nine deep models on the internal dataset (CirCor, patient level) and the two external datasets (CinC 2016 and Yaseen 2018, recording level). References [41,42] are cited in the Supplementary Materials.

## Abbreviations

The following abbreviations are used in this manuscript:

1D-CNN	One-dimensional convolutional neural network
2D-CNN	Two-dimensional convolutional neural network
AP	Average precision
AUROC	Area under the receiver operating characteristic curve
BA	Balanced accuracy
CI	Confidence interval
CinC	PhysioNet/Computing in Cardiology Challenge 2016 dataset
CLAIM	Checklist for Artificial Intelligence in Medical Imaging
CNN-BiLSTM	Convolutional bidirectional long short-term memory network
CWT	Continuous wavelet transform
DANN	Domain-adversarial neural network
ECE	Expected calibration error
GPU	Graphics processing unit
Grad-CAM	Gradient-weighted class activation mapping
Hybrid-KAN	Hybrid raw-waveform and spectrogram network with a Kolmogorov–Arnold network head
Hybrid-MLP	Hybrid raw-waveform and spectrogram network with a multilayer perceptron head
KAN	Kolmogorov–Arnold network
MaxViT	Multi-axis vision transformer
MC	Monte Carlo
MLP	Multilayer perceptron
OOD	Out-of-distribution
PCA	Principal component analysis
PCG	Phonocardiogram
ResNet1D	One-dimensional residual network
Sens	Sensitivity
Spec	Specificity
SVM-RBF	Support vector machine with a radial basis function kernel
TRIPOD+AI	Transparent Reporting of a multivariable prediction model for Individual Prognosis Or Diagnosis, updated for Artificial Intelligence
t-SNE	t-distributed stochastic neighbor embedding
