# Supplementary material for: Cross-Dataset Generalization in Phonocardiogram Murmur Classification: A Benchmark Study of Domain-Conditional Performance and the Role of Multi-Representation Fusion
Source: J Clin Med. 2026 Oct 17;15(20):8058. doi: 10.3390/jcm15208058 (PMC13706609; doi:10.3390/jcm15208058)
Supplement: Supplementary file 1 [file jcm-15-08058-s001.zip › jcm-4481322-supplementary.pdf]

Supplementary Materials

Cross-Dataset Generalization in Phonocardiogram Murmur Classification: A Benchmark Study of Domain-Conditional Performance and the Role of Multi-Representation Fusion

Hüseyin Kutlu [1,2] and Cemil Çolak [2,*]

[1] Department of Biostatistics and Medical Informatics, Faculty of Medicine, Adıyaman University, 02040 Adiyaman, Türkiye; hkutlu@adiyaman.edu.tr
[2] Department of Biostatistics and Medical Informatics, Faculty of Medicine, İnönü University, 44280 Malatya, Türkiye
[*] Correspondence: cemil.colak@inonu.edu.tr

Reference numbers refer to the reference list of the main article.

Table S1. Common preprocessing and time–frequency representation parameters applied identically to all three datasets. No data augmentation or synthetic data were used.

Parameter	Value
Target sampling rate	2000 Hz (common rate; CinC native)
Band-pass filter	Butterworth, 4th order, 25–400 Hz, zero-phase (filtfilt)
Spike/artefact handling	IQR-based clipping, k = 3.0 × IQR
Amplitude normalization	Per-recording z-score (zero mean, unit variance)
Segment window	5.0 s fixed length
Segment hop / overlap	2.5 s hop (50% overlap); zero-padding if shorter than one window
Raw waveform length	10 000 samples per segment (5 s × 2000 Hz)
Mel spectrogram: n_fft	1024
Mel spectrogram: hop length	50 samples (25 ms)
Mel spectrogram: window length	100 samples (50 ms)
Mel spectrogram: n_mels	64
Mel spectrogram: f_min / f_max	25 Hz / 400 Hz
Mel spectrogram: power, scale	power = 2.0; log scale (power-to-dB, ref = max)
CWT scalogram: output shape	64 frequency scales × 201 time frames
CWT wavelet family	Morlet (ssqueezepy), 8 voices per octave (nv = 8)
CWT analysis band / pooling	25–400 Hz signal band; magnitude average-pooled to 64 frequency bins × 201 time frames, log1p-compressed, per-sample z-scored
Random seed	42

Mel spectrograms were computed with librosa and CWT scalograms with ssqueezepy (Morlet wavelet); PyWavelets was used only for the DWT-based features of the classical baselines. The two external datasets were processed with the identical pipeline; no external data informed any preprocessing choice.

Table S2. Training configuration shared by the deep models, and the additional settings for the domain-adaptation and uncertainty analyses. All models were trained only on the CirCor training data with five-fold patient-disjoint cross-validation.

Setting	Value
Optimizer	AdamW
Learning rate	3×10^{-4}
Weight decay	1×10^{-4}

Setting	Value
Learning-rate schedule	Linear warm-up (3 epochs) then cosine decay
Batch size	64
Maximum epochs	30
Early stopping	On EMA (window 3) of validation AUROC; patience 8 epochs
Model selection	Checkpoint at best validation AUROC
Loss	Class-weighted cross-entropy, label smoothing 0.05
Class-imbalance handling	Weighted random sampler (β = 0.5) + class weights
Gradient clipping	Max-norm 0.5
Cross-validation	5-fold, patient-disjoint, stratified by murmur class
External evaluation	5-fold ensemble (mean of fold probabilities)
Random seed	42
DANN gradient-reversal λ schedule	Delayed sigmoid ramp 0→1: $\lambda = 2/(1+e^{-5p}) - 1$, p = max(0,(epoch_fraction−0.2)/0.8); first 20% of epochs λ≈0; domain-classifier LR = 2× feature-extractor LR
MC-dropout forward passes	20 (normalization layers fixed to eval, dropout active)
Deep-ensemble size	5 models (seeds 42, 142, 242, 342, 442); uncertainty benchmark evaluated across all 5 folds
Multi-architecture ensemble	Soft voting; two configurations (all models; balanced subset)

Hyperparameters were identical across the deep architectures unless dictated by the architecture itself; no per-dataset tuning was performed, and the external datasets were never used for model selection. The two classical baselines used: SVM with an RBF kernel (C = 10, gamma = scale, balanced class weights) and a random forest (300 trees, no depth limit, balanced class weights, seed 42), both trained on a 63-dimensional feature vector (MFCC with deltas, spectral centroid/bandwidth/rolloff, zero-crossing rate, DWT db4 level-5 band energies, and time-domain statistics).

Table S3. Software library versions, hardware, and reproducibility settings.

Component	Version / specification
Python	3.13.2
PyTorch	2.10.0 (CUDA 12.8)
torchvision	0.25.0
librosa	0.11.0
scikit-learn	1.8.0
NumPy / SciPy	2.5.1 / 1.16.3
PyWavelets / ssqueezepy	1.8.0 / 0.6.6
timm	1.0.22
mamba-ssm	2.3.0
einops / pandas	0.8.2 / 2.3.3
GPU	NVIDIA RTX 6000 Ada Generation (48 GB)
System memory / CPU	128 GB RAM; Intel Core i9 (14th generation)
Random seed	42 (base seed; deep-ensemble seeds in Table S2)
Approx. training runtime	CNN-BiLSTM ≈ 3 min; PCG-MambaConformer ≈ 14 min (full 5-fold model, single GPU)
Approx. external inference runtime	A few seconds per external dataset (5-fold ensemble)

Table S4. Number of recordings and 5-second segments per dataset after preprocessing. CirCor served as the internal training source (five-fold cross-validation); CinC 2016 and Yaseen 2018 were used only for zero-shot external evaluation.

Dataset	Recordings	Segments	Class distribution
CirCor (internal)	2531	19 460	Absent 1906 / Present 494 / Unknown 131 (753 patients)
CinC 2016 (external)	3240	24 450	Normal 2575 / Abnormal 665
Yaseen 2018 (external)	1000	1000	Normal 200 / Abnormal 800

CirCor counts correspond to the cross-validation training set (patient-disjoint, stratified by murmur). The "unknown" murmur label (131 recordings, 5.2% of CirCor) denotes recordings in which the annotator could not confidently exclude a murmur; these were grouped with "present" (abnormal) as the conservative screening choice. Yaseen recordings are short, yielding one 5-second segment each; CinC recordings vary in length (5.3 to 122.0 s; median 20.8 s) and are drawn from six sub-databases, yielding 24 450 segments from 3240 recordings.

Table S5. Effect of excluding the 131 unknown-murmur recordings from training. Two representative models were retrained on Present-versus-Absent recordings only and re-evaluated, without target-domain fine-tuning, on both external datasets. Values are AUROC with 95% bootstrap confidence intervals; the original column reproduces the values obtained with the conservative grouping (unknown treated as abnormal).

Model / Target	Original	Excluding unknown (95% CI)	Difference
CNN-BiLSTM — Internal	0.903	0.965 [0.947–0.980]	+0.062
CNN-BiLSTM — CinC	0.727	0.662 [0.641–0.681]	−0.065
CNN-BiLSTM — Yaseen	0.806	0.483 [0.448–0.519]	−0.323
PCG-MambaConformer — Internal	0.862	0.948 [0.922–0.970]	+0.086
PCG-MambaConformer — CinC	0.601	0.447 [0.421–0.474]	−0.154
PCG-MambaConformer — Yaseen	0.985	0.893 [0.873–0.912]	−0.092

Across both models, excluding the unknown recordings raised the internal AUROC but lowered AUROC on both external targets. This indicates that the grouping influenced the absolute external values; the direction of the effect (better internal, worse external) is consistent with the study's central finding that internal accuracy is a weak indicator of external behaviour. PCG-MambaConformer retained balanced transfer to the homogeneous target without the unknown recordings (Yaseen AUROC 0.893).

Table S6. TRIPOD+AI reporting map.

Reporting follows the TRIPOD+AI statement [41], which supersedes TRIPOD 2015.

Item	Reporting element (summarized)	Where reported	Status / comments
**1**	Title identifies the work as developing and externally evaluating a multivariable prediction model, the target population, and the predicted outcome.	Title	Reported. Title states cross-dataset PCG murmur classification (normal vs abnormal).
**2**	Structured abstract (background/objectives, methods, results, conclusions) reporting at least one discrimination metric with uncertainty.	Abstract	Reported. AUROC with sensitivity/specificity; see TRIPOD+AI for Abstracts.
**3**	Background: rationale, clinical context, and reference to existing models/approaches.	§1 Introduction	Reported. Auscultation limits, prior PCG ML, dataset-shift problem.
**4**	Objectives: specific study objectives, including intended use.	§1 (final paragraph)	Reported. Threefold aims (compare, adapt/quantify, characterize).
**5**	Source of data and study design (development vs evaluation; key dates; zero-shot external design).	§2.1; Table S4	Reported. CirCor internal; CinC 2016 and Yaseen 2018 external (zero-shot).

Item	Reporting element (summarized)	Where reported	Status / comments
**6**	Participants/setting: eligibility, recording setting, and population of each dataset.	§2.1	Reported. Pediatric CirCor; adult/mixed CinC; five-class Yaseen.
**7**	Data preparation: preprocessing and how signals/representations were derived.	§2.1, §2.2; Table S1	Reported. Resampling, band-pass, 5 s segments; raw/mel/CWT.
**8**	Outcome: definition and how the label was determined; any grouping decisions.	§2.1; §3.6	Reported. Binary label; unknown grouped as abnormal (conservative); see §3.6.
**9**	Predictors/features used by the models.	§2.2; Table S2	Reported. 63-dim features (classical); raw/mel/CWT (deep).
**10**	Sample size and event counts.	Table S4	Reported. Recording/segment counts per dataset. No a priori power calculation (fixed public datasets); stated.
**11**	Missing data: how handled.	§3.6; Table S5	N/A for classic missingness (complete recordings). Ambiguous 'unknown' labels handled via sensitivity analysis.
**12**	Analytical methods: model types/architectures compared.	§2.2	Reported. 11 approaches incl. four-branch PCG-MambaConformer.
**13**	Model training: CV scheme, optimizer, class-imbalance handling, hyperparameters.	§2.3; Table S2	Reported. 5-fold patient-disjoint CV; AdamW; class weighting; fixed hyperparameters.
**14**	Model output and how a prediction is produced (threshold; ensembling).	§2.3	Reported. Predicted probability; 0.5 threshold; 5-fold ensemble externally.
**15**	Performance measures and how estimated (with uncertainty).	§2.3	Reported. AUROC (primary), average precision, sens/spec at three thresholds; bootstrap & Wilson CIs; DeLong; Holm.
**16**	Model evaluation: internal and external validation; leakage checks.	§2.3, §2.5; §3.6	Reported. Internal CV (patient level) + zero-shot external (recording level); record-overlap leakage check; reciprocal-direction transfer (Table S8).
**17**	Fairness/heterogeneity of performance across subgroups.	§2.5; §3.6	Partially addressed via per-pathology transfer (AS/MR/MS/MVP) and reciprocal pediatric/adult transfer (Table S8). No demographic fairness analysis (data-limited); stated.
**18**	Open science: data, code, protocol, reproducibility.	§2.6; Data Availability	Reported. Public datasets; open GitHub repo; fixed seed.
**19**	Patient and public involvement.	—	N/A. Secondary use of fully anonymized public datasets; no PPI.
**20**	Participants/data flow: counts and class distribution.	Table S4	Reported.
**21**	Model specification: final architecture and components.	§2.2, §3; GitHub	Reported. Four-branch fusion; branch definitions; open code.

Item	Reporting element (summarized)	Where reported	Status / comments
**22**	Model performance: internal and external results with uncertainty.	§3.1–3.8; Tables 1–4; Figs 1–9	Reported.
**23**	Model updating/adaptation: domain adaptation and ensembling results.	§3.5, §3.7	Reported. DANN (conditional effect); multi-architecture ensembling; threshold recalibration (§3.4, Table 3).
**24**	Additional analyses: ablation, uncertainty, feature-space, saliency.	§3.7–3.8	Reported. Branch ablation; UQ benchmark; t-SNE/silhouette; Grad-CAM; operating-point transfer analysis.
**25**	Interpretation: overall interpretation, in context of objectives and prior evidence.	§4 Discussion	Reported. Homogeneity-dependent transfer; internal≠external.
**26**	Limitations: sample, design, generalizability.	§4	Reported. Single internal dataset; binary framing; two external sets; no calibration on labeled target samples; no prospective test.
**27**	Implications and next steps for research/deployment.	§4	Reported. Multi-source training; target-aware adaptation; prospective evaluation.

Table S7. CLAIM (2024 update) reporting map.

Reporting of the image-based (CWT scalogram) and saliency (Grad-CAM) components follows the Checklist for Artificial Intelligence in Medical Imaging (CLAIM), 2024 update [42]. Because the analyzed images are signal-derived scalograms rather than acquired clinical radiology images, acquisition- and radiologist-read–specific items are marked N/A with a brief reason.

Item	Reporting element (summarized)	Where reported	Status / comments
**1**	Identifies use of AI/ML methods in the title.	Title	Reported (deep-learning benchmark).
**2**	Structured summary of design, methods, results.	Abstract	Reported.
**3**	Scientific/clinical background.	§1	Reported.
**4**	Study objectives and hypotheses.	§1	Reported.
**5**	Study design (prospective/retrospective).	§2.1	Retrospective secondary analysis of public datasets.
**6**	Study goal (e.g., classification, detection).	§1, §2	Binary murmur classification; cross-dataset transfer.
**7**	Data sources.	§2.1; Table S4	CirCor, CinC 2016, Yaseen 2018.
**8**	Eligibility / inclusion criteria for data.	§2.1	Dataset-defined; all usable recordings.
**9**	Data preprocessing (incl. image/scalogram generation).	§2.1; Table S1	Resampling, band-pass, 5 s segments, CWT scalogram params.
**10**	Selection of data subsets.	§2.1; Table S4	CV training set; external evaluation sets.

Item	Reporting element (summarized)	Where reported	Status / comments
**11**	De-identification of data.	Data Availability; IRB statement	Public, fully anonymized datasets.
**12**	Handling of missing data.	§3.6	N/A (complete recordings); ambiguous labels via sensitivity analysis.
**13**	Definition and rationale of reference standard (ground truth).	§2.1	Expert murmur annotations from source datasets (established benchmarks).
**14**	Source of ground-truth annotations.	§2.1	Original dataset annotators (e.g., CirCor expert labels).
**15**	Annotation tools / rater qualifications.	§2.1	Inherited from source datasets; not re-annotated (stated as N/A for local reads).
**16**	Inter-rater variability of ground truth.	—	N/A. Labels inherited from public datasets.
**17**	Data partitions and level defined.	§2.3; §3.6	5-fold patient-disjoint CV (internal); zero-shot external; record-level.
**18**	Independence of partitions / leakage prevention.	§3.6	Record-identifier overlap check; patient-disjoint folds.
**19**	Model type and architecture.	§2.2	11 approaches; four-branch PCG-MambaConformer.
**20**	Software libraries, frameworks, versions.	§2.6; Table S3	PyTorch, timm, mamba-ssm, librosa, etc.
**21**	Initialization / pretraining of parameters.	§2.2	timm backbones; official Mamba implementation.
**22**	Training approach (optimizer, LR, epochs, batch).	§2.3; Table S2	AdamW; cosine schedule; class weighting.
**23**	Hyperparameter selection method.	§2.3; Table S2	Fixed across architectures; no per-dataset tuning; external never used for selection.
**24**	Evaluation metrics.	§2.3	AUROC, average precision, sensitivity, specificity, balanced accuracy.
**25**	Statistical measures of significance / uncertainty.	§2.3	Bootstrap & Wilson CIs; DeLong; Holm–Bonferroni.
**26**	Robustness / sensitivity analyses.	§3.4, §3.6, §3.7	Ablation; unknown-label exclusion; reciprocal transfer; threshold sensitivity; two state-space implementations.
**27**	Explainability / interpretability methods.	§2.5; §3.8	t-SNE + silhouette; Grad-CAM on CWT branch.
**28**	Flow of data / participant counts.	Table S4	Reported.
**29**	Model performance results.	§3; Tables 1–4; Figs 1–9	Reported.
**30**	Failure/limitation analysis of the model.	§3.1–3.4, §3.7	Operating-point collapse; discrimination versus threshold-transfer failure separated; heterogeneous-target failure documented.

Item	Reporting element (summarized)	Where reported	Status / comments
**31**	Study limitations.	§4	Reported.
**32**	Implications for clinical practice / deployment.	§4	Reported.
**33**	Registration.	—	N/A. Secondary analysis of public data; not registered.
**34**	Funding and conflicts of interest.	Funding; Conflicts of Interest	Reported.
**35**	Code and data availability.	Data Availability; GitHub	Reported.

Table S8. Reciprocal (reverse-direction) cross-dataset transfer. Two representative models were retrained from scratch on PhysioNet/CinC 2016 with record-disjoint five-fold cross-validation and evaluated, without any target-domain fine-tuning, on the pediatric CirCor data (patient level) and on Yaseen 2018 (recording level). Forward-direction values (CirCor to target, Tables 1 and 2) are reproduced for comparison. Sensitivity and specificity are at the 0.5 threshold.

Model	Direction	Training source	Evaluation target	AUROC (95% CI)	Sens / Spec
CNN-BiLSTM	Forward	CirCor	CinC 2016	0.727 [0.708–0.746]	0.502 / 0.777
CNN-BiLSTM	Forward	CirCor	Yaseen 2018	0.806 [0.779–0.833]	0.471 / 1.000
CNN-BiLSTM	Reverse	CinC 2016	CirCor (patient level)	0.677 [0.633–0.718]	0.736 / 0.498
CNN-BiLSTM	Reverse	CinC 2016	Yaseen 2018	0.926 [0.908–0.944]	1.000 / 0.075
PCG-MambaConformer	Forward	CirCor	CinC 2016	0.601 [0.576–0.624]	0.198 / 0.932
PCG-MambaConformer	Forward	CirCor	Yaseen 2018	0.985 [0.977–0.992]	0.718 / 0.995
PCG-MambaConformer	Reverse	CinC 2016	CirCor (patient level)	0.612 [0.565–0.657]	0.411 / 0.710
PCG-MambaConformer	Reverse	CinC 2016	Yaseen 2018	0.942 [0.924–0.958]	0.894 / 0.875

Yaseen 2018 was not used as a training source in either direction. Its five classes are specific valvular lesions, so a Yaseen-trained model would be a lesion detector rather than a general abnormality detector, and its transfer to CirCor would measure label mismatch rather than domain shift. Because CinC 2016 provides no patient identifiers, the reverse-direction folds were record-disjoint rather than patient-disjoint. The forward and reverse barriers are of comparable magnitude, indicating that the difficulty is attached to the heterogeneous dataset itself rather than to the pediatric origin of the training data alone.

Table S9. Operating-point analysis on the internal CirCor dataset (patient level; abnormal prevalence 0.262). The source-derived Youden threshold and the target-optimal threshold coincide here by construction, since both are computed on the same out-of-fold predictions; this serves as an internal validity check for the threshold-transfer analysis in Table 3. AP = average precision; BA = balanced accuracy.

Approach	AP	Sens / Spec @ 0.5	Sens / Spec @ Youden	BA (0.5 –> Youden)	Source threshold
1D-CNN	0.683	0.594 / 0.847	0.868 / 0.642	0.721 –> 0.755	0.171
2D-CNN	0.721	0.736 / 0.712	0.614 / 0.878	0.724 –> 0.746	0.671
ResNet1D	0.735	0.594 / 0.887	0.558 / 0.944	0.740 –> 0.751	0.593
CNN-BiLSTM	0.836	0.746 / 0.899	0.746 / 0.905	0.823 –> 0.825	0.507
Hybrid-MLP	0.820	0.609 / 0.957	0.716 / 0.926	0.783 –> 0.821	0.411
Hybrid-KAN	0.831	0.721 / 0.903	0.812 / 0.835	0.812 –> 0.823	0.392
EfficientNet-B0	0.791	0.482 / 0.986	0.746 / 0.867	0.734 –> 0.807	0.301
MaxViT	0.781	0.690 / 0.887	0.777 / 0.811	0.789 –> 0.794	0.394
PCG-MambaConformer	0.782	0.650 / 0.915	0.716 / 0.881	0.783 –> 0.799	0.402

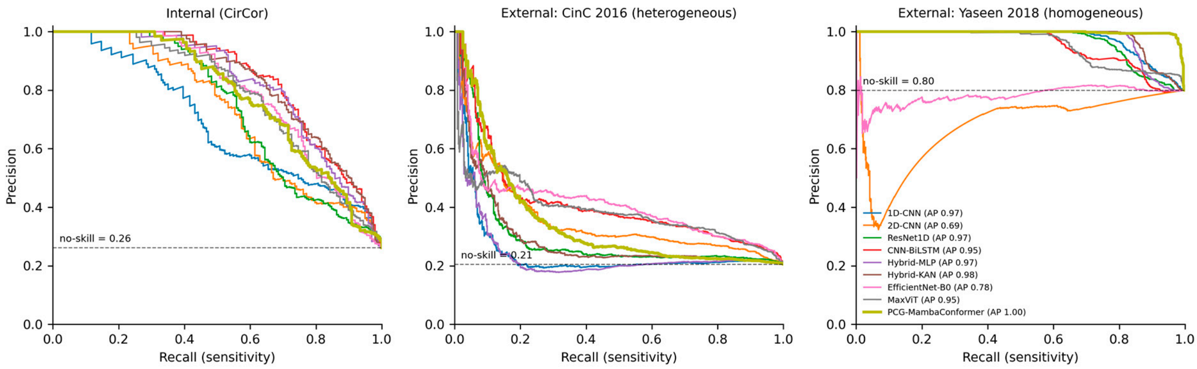

Figure S1. Precision–recall curves for the nine deep models on the internal dataset (CirCor, patient level) and the two external datasets (CinC 2016 and Yaseen 2018, recording level). The dashed horizontal line in each panel marks the no-skill baseline, which equals the abnormal prevalence of that dataset (0.262, 0.205 and 0.800, respectively). Average precision values are given in the legend and in Table 3. PCG-MambaConformer is drawn with a heavier line.
